# Targeting TDP-43 Proteinopathy in hiPSC-Derived Mutated hNPCs with Mitoxantrone Drugs and miRNAs

**DOI:** 10.3390/pharmaceutics17040410

**Published:** 2025-03-25

**Authors:** Uzair A. Ansari, Ankita Srivastava, Ankur K. Srivastava, Abhishek Pandeya, Pankhi Vatsa, Renu Negi, Akash Singh, Aditya B. Pant

**Affiliations:** 1 Systems Toxicology Group, FEST Division, CSIR-Indian Institute of Toxicology Research, Vishvigyan Bhawan, 31, Mahatma Gandhi Marg, Lucknow 226001, Uttar Pradesh, India; ansari.uzair009@gmail.com (U.A.A.); srivastav.ankita@hotmail.com (A.S.); ank.biotech@hotmail.com (A.K.S.); pandeyaabhishek@hotmail.com (A.P.); vatsapankhi@gmail.com (P.V.); renu20negi@gmail.com (R.N.); singh.akash1398@hotmail.com (A.S.); 2 Academy of Scientific and Innovative Research (AcSIR), Ghaziabad 201002, Uttar Pradesh, India

**Keywords:** ALS model, miRNA, high-throughput screening, TDP-43, mitoxantrone, human neural progenitor cells (hNPCs), hiPSCs

## Abstract

**Background/Objectives:** TDP-43 mutation-driven Amyotrophic Lateral Sclerosis (ALS) motor neuron disease is one of the most prominent forms (approximately 97%) in cases of sporadic ALS. Dysfunctional autophagy and lysosomal function are the prime mechanisms behind ALS. Mitoxantrone (Mito), a synthetic doxorubicin analog, is an inhibitor of DNA and RNA synthesis/repair via intercalating with nitrogenous bases and inhibiting topoisomerase II. The therapeutic potential of miRNAs associated with disease conditions has also been reported. This study explores the therapeutic potential of Mito along with miRNAs against mutated TDP-43 protein-induced proteinopathy in human-induced pluripotent stem cell (hiPSC)-derived human neural progenitor cells (hNPCs). **Methods**: HiPSCs mutated for TDP-43 were differentiated into hNPCs and used to explore the therapeutic potential of Mito at a concentration of 1 μM for 24 h (the identified non-cytotoxic dose). The therapeutic effects of Mito on miRNA expression and various cellular parameters such as mitochondrial dynamics, autophagy, and stress granules were assessed using the high-throughput Open Array technique, immunocytochemistry, flow cytometry, immunoblotting, and mitochondrial bioenergetic assay. **Results**: Mutated TDP-43 protein accumulation causes stress granule formation (G3BP1), mitochondrial bioenergetic dysfunction, SOD1 accumulation, hyperactivated autophagy, and ER stress in hNPCs. The mutated hNPCs also show dysregulation in six miRNAs (miR-543, miR-34a, miR-200c, miR-22, miR-29b, and miR-29c) in mutated hNPCs. A significant restoration of TDP-43 mutation-induced alterations could be witnessed upon the exposure of mutated hNPCs to Mito. **Conclusions**: Our study indicates that miR-543, miR-29b, miR-22, miR-200c, and miR-34a have antisense therapeutic potential alone and in combination with Mitoxantrone.

## 1. Introduction

Amyotrophic Lateral Sclerosis (ALS) is a progressive and often late-diagnosed motor neuron degeneration disease that leads to the death of patients within 2–3 years post-diagnosis due to respiratory failure [1]. A meta-analysis estimated the global burden of ALS by the prevalence of ALS, from 1.57 to 9.62 cases per 100,000 population, and incidence, from 0.42 to 2.76 cases per 100,000 population up to the year 2021 [2]. Among more than 20 gene mutations associated with ALS, five (*C9orf72*, *SOD1*, *TARDBP*, *FUS*, and *TBK1*) are the most common genetic causes. TDP-43, a protein encoded by the *TARDBP* gene, has been found to be an abnormal aggregate in the cytoplasm of motor neurons in over 95% of sporadic ALS cases, making it a key pathological hallmark of the disease. It is a nuclear RNA-binding protein predominantly found in the nucleus, which mislocalizes to the cytoplasm to form aggregates upon mutation in ALS [3]. This accumulation of mutated TDP-43 proteins in ALS creates chronic stress and induces pathologically stable inclusions, i.e., stress granules. These static pathological granules further trigger the signaling for the pro-death mechanism of motor neurons in the ALS condition [4]. The induction of these granules, as a response to chronic stress, may also be a seeding event for dysfunction in vital cellular mechanisms such as mitochondrial function, the cellular proteostasis system (ubiquitin proteosome pathways, autophagy, the endoplasmic reticulum system, and chaperons), cytoskeletal disturbances, and axonal transport defects [5]. The available research also suggests that the overexpressed mutant TDP-43-driven formation of stress granules interacts with and inhibits miRNA biogenesis and its regulatory function [6]. A cellular-model-based miRNA profiling study has identified 65 cytoplasmic TDP43-associated miRNAs that may have a pathogenic role in modifying the downstream expression of genes and pathways in ALS [7].

Human-derived induced pluripotent cells (hiPSCs) have emerged as high-throughput in vitro tools for disease modeling and drug discovery [8]. The reprogramming of human somatic cells into hiPSCs and the downstream desired cellular fate have enabled scientists to learn about the neurochemistry behind rare progressive diseases and explore their druggable targets [9]. We previously identified SOD1L39R mutation in ALS patient fibroblasts with the help of gene sequencing. We demonstrated that hyperactivated astrocytes contribute to non-cell autonomous neurotoxicity in MNs in the SOD1L39R-linked ALS model [10]. They are important in cellular ALS models at the initiation level for motor neuron differentiation (i.e., mutated hNPCs). Further, integrating high-throughput miRNA profiling with hiPSC-based disease models offers a robust platform for identifying key regulatory biomolecules and elucidating the mechanisms underlying disease etiology, potentially unveiling novel therapeutic targets [11]. Several Food and Drug Administration (FDA)-approved disease-modifying therapies (Riluzole, Edaravone, AMX0035, Nuedexta, and Tofersen) are already on the market, acting against various mutations, and many are under active investigation in clinical trials [12]. Despite there being few drugs specific to TDP-43-specific proteinopathies being under clinical trial, we still lack an FDA-approved and TDP-43-directed therapy to slow or reverse the ALS disease [7]. Mitoxantrone (Mito) is a synthetic nucleic acid and intercalating agent that can directly engage with DNA/RNA. It is used as a drug for impeding DNA synthesis, DNA repair, and topoisomerase II activity in tumor cells. Mitoxantrone neurotoxicity remains inadequately explored despite its long-standing application in cancer therapy and, more recently, in the treatment of multiple sclerosis. However, chemotherapy-induced cognitive dysfunction substantially impacts the post-treatment quality of life in patients. It is also an immunomodulatory and immunosuppressive drug used to treat multiple sclerosis [13]. Therefore, the present study aims to explore the therapeutic potential of Mito against TDP-43-induced proteinopathy in the ALS condition. Here, we report for the first time the in vitro efficacy of Mito in mitigating key pathological abnormalities, such as mitochondrial dysfunction, autophagy, ER stress, and stress granules, associated with ALS. This study also proposes dysregulated miRNAs in generated mutated NPCs using the in vitro ALS model. It also functions as an immunomodulatory and immune-suppressive drug in treating multiple sclerosis. In the present investigations, we have explored the therapeutic potential of Mito against TDP-43-induced proteinopathy in ALS conditions. Further, in silico simulations have also been conducted to assess the target-specific therapeutic potential of miRNAs identified as associated with TDP-43-mediated ALS conditions. These miRNAs are also shown as a potential target of the Mito drug to restore pathological symptoms at the cellular level. We anticipate the protective effect of Mitoxantrone and the role of miRNAs, which can be used as antisense therapeutic targets for ALS cure/management by targeting key underlying pathology mechanisms.

## 2. Materials and Methods

The human iPSC cell line (Episomal human iPSCs, Cat#A18945) was commercially purchased from Gibco™, Thermofisher Scientific, Waltham, MA, USA. Culture mediums like the Essential 8 medium (Cat# A1517001); DMEM/F12 medium (Cat# 12500062); Neurobasal Medium (Cat# 21103049); StemFlex™ Medium (Cat # A3349401); several reagents like the N2 supplement (Cat# 17502048) and B27 Supplement (Cat# 17504044); StemPro Accutase™ (Cat# A1110501); KnockOut™ SR (Cat# 10828010); 4-well chamber slides (Cat # 177399) and D-PBS (Cat# 21600010); GlutaMAX™ (Cat# 35050061); High-Capacity cDNA Reverse Transcription Kit (Cat# 4368814); TrueCut™ Cas9 Protein (A36497) GeneArt™ Genomic Cleavage Detection Kit (A24372); and Neon™ Transfection System 10 μL Kit (Cat # MPK1096) were procured from Thermo Fisher Scientific (Waltham, MA, USA). The necessary growth factors like Recombinant Human FGF-basic 154 a.a (Cat# 100-18B), Human BDNF (Cat# 45002), Human NT-3 (Cat #AF-45003), and Human EGF (AF-10015) were procured from PeproTech Inc., Cranbury, NJ, USA. ROCK inhibitor-Y-27632 (Cat# 1254), SB431542 (Cat# 1614), and Dorsomorphin (Cat# 3093) were procured from Tocris Bioscience, Bristol, UK. LDEV-free Corning^®^ Matrigel^®^ Basement Membrane Matrix (Cat# 354234), LDEV-free Corning^®^ Matrigel^®^ Growth Factor Reduced (GFR) Basement Membrane Matrix (Cat# 356230), and ultra-low attachment 6-well plates (Cat# 3271) were obtained from Corning^®^, NY, USA. JC-1 Dye (Cat#13168) and ER Tracker (Cat# E34250) were procured from Thermo Fisher Scientific, Waltham, MA, USA (Cat# E34250). Seahorse XF HS Mini and XFp analyzers (Cat#130010-100) and Seahorse XFp Cell Mito Stress Test Kit (Cat#103010-100) were procured from Agilent (Hachioji, Tokyo). Mitoxantrone dihydrochloride (Cat#M6545) was procured from Sigma (Kawasaki, Japan). Vectashield Antifade Mounting Medium with DAPI (Cat#H-1200-10) and primary antibodies, i.e., SOD1 (Cat#ab51254), G3BP (Cat#ab181149), Nestin (Cat#ab22035), LC3-B (Cat#ab221794), LAMP-1 (Cat#ab25630), TDP43 (Cat#ab133547), SQSTM1 (Cat#ab56416), SOX2 (Cat#ab137385), HSP70 (Cat#ab181606), and Pax6 (Cat#ab195045), were procured from Abcam (Cambridge, UK). Oct-4A (C52G3) (Cat#75463S) and Nanog (1E6C4) were procured from Cell Signaling Technology (CST, Danvers, MA, US). All secondary antibodies, i.e., Alexa Fluor^®^ 488 goat anti-rabbit (Cat#A11034), goat anti-mouse 488 (Cat#M32501), Alexa Fluor^®^ 546 goat anti-rabbit (Cat#A11010), Alexa Fluor^®^ 546 goat anti-mouse (Cat#A11003), and Alexa Fluor^®^ 568 goat anti-mouse (Cat#A11031), were purchased from Thermo Fisher Scientific, Waltham, MA, USA.

### 2.1. Cultivation and Characterization of Human-Induced Pluripotent Stem Cells (hiPSCs)

hiPSCs were procured from Gibco™ and cultured in accordance with the standardized protocol previously established by our research group [10]. Briefly, hiPSCs were seeded in 60 mm tissue culture dishes coated with the corning Matrigel basement matrix and maintained in pre-warmed Essential 8 medium (E8 medium). Upon reaching approximately 80% confluence, the cells were routinely passaged and cryopreserved at a split ratio of 1:3 to 1:4. The cultures were maintained in a humidified incubator at 37 °C with 5% CO_2_. Following every fifth passage, colonies exceeding 80% confluence were evaluated for normal morphology and the expression of stemness and potency markers. The characterized and purified hiPSCs population was subsequently utilized for differentiation into neural progenitor cells (hNPCs) for all experimental procedures.

Immunocytochemistry was employed to analyze hiPSCs. The hiPSCs were plated on a 4-well chamber slide coated with Matrigel and maintained in an E8 medium. Twenty-four hours after plating, the cells were fixed with 4% paraformaldehyde (PFA) for twenty minutes at room temperature and then permeabilized with 0.5% Triton X-100 in phosphate-buffered saline (PBS) for 10–15 min on a rocker shaker at room temperature. The cells were subsequently rinsed with PBS and treated with a blocking solution of 0.2% Triton X-100 and 0.1% bovine serum albumin (BSA) for one hour at room temperature. After blocking, the cells were incubated with primary antibodies specific to the pluripotency markers SOX2 and OCT4 (dilution 1:200) overnight at 4 °C. After washing with PBS, the cells were incubated with Alexa Fluor-488- and Alexa Fluor-595-conjugated secondary antibodies (dilution 1:3000) for two hours at 25 °C. A final wash with PBS was performed. The cells were stained with 4′,6-diamidino-2-phenylindole (DAPI) to visualize nuclei. Images were captured using an Evos Auto FL fluorescent microscope [14].

### 2.2. Development of hiPSCs Harboring TDP-43 Genetic Mutation

The mutation in hiPSCs was achieved using the CRISPR/Cas-9 gene-editing technology in conjunction with pre-designed guide RNA (gRNA), following the protocol developed in our laboratory [10]. Briefly, hiPSCs were transfected via an electroporation method using the Invitrogen™ Neon™ Transfection System MPK5000 and Neon™ Transfection System 10 μL Kit (Cat # MPK1096). This system facilitated the delivery of a TDP-43-specific gRNA with the sequence 5′-ACATCCGATTTAATAGTGTT-3′, as per the manufacturer’s instructions. Post-electroporation, the hiPSCs were cultured in 12-well plates coated with the Matrigel basement membrane matrix. The cells were maintained in a Stem Flex medium supplemented with a ROCK inhibitor (10 μM) and incubated at 37 °C in a humidified environment containing 5% CO_2_. The GeneArt Genomic Cleavage Detection kit and cellular characterization protocol (Immunocytochemistry) were used to assess the transfection’s efficacy of generated mutations.

### 2.3. Generation, Characterization, and Metabolic Flux Analysis of Normal and Mutated hiPSC-Derived Neural Progenitor Cells

Characterized hiPSC lines, both normal and mutated, were cultured in Essential 8 medium on Matrigel-coated 60 mm dishes. The cells were incubated at 37 °C with a 5% CO_2_ atmosphere to promote the best growth conditions. Normal and mutated human neural progenitor cells (hNPCs) were obtained by manually selecting and transferring hiPSC colonies into ultra-low attachment plates to facilitate embryoid body (EB) formation. These EBs were then cultured on Matrigel-coated plates to develop into hNPCs, and immunocytochemical analysis was performed according to a previously documented protocol [10]. The primary antibodies against pluripotency markers and progenitor markers PAX6 and Nestin (dilution 1:200) were used for ICC.

Further, hNPCs were analyzed using a BD FACS Canto-II flow cytometer (Becton Dickinson, San Jose, CA, USA). During the analysis, the cells were treated with specific surface markers, SSEA-4 and CD133, conjugated to Alexa Fluor 647 and 488 fluorescent dyes, respectively. After incubation, the cells underwent three washes with PBS and were subsequently suspended in 0.5 mL PBS solution. A control group of cells, not exposed to the antibodies but maintained under the same conditions, was used for comparison. Standardized acquisition protocols were applied during the process. Cell populations were initially gated based on the characteristics of forward and side scatter (FSC vs. SSC). For each sample, 10,000 events were recorded at a medium flow rate. The percentage of cell populations was analyzed using FACSDiva Version 6.1.2 and FlowJo V10 software (USA).

Bioenergetic profiles and oxygen consumption rates (OCR) were assessed using the Seahorse XFp Analyzer (Agilent, Santa Clara, CA, USA) for both normal (N-hiPSCs) and their mutated counterparts (M-hiPSCs). Cells from each group were plated on Seahorse XFp mini plates to undergo metabolic flux analysis. Prior to analysis, the standard culture medium in the mini plates was substituted with an unbuffered Seahorse XF base medium (Dulbecco’s Modified Eagle Medium supplemented with 10 mM glucose, 1 mM sodium pyruvate, and 2 mM Glutamax, adjusted to pH 7.4). Following a 1 h incubation at 37 °C, the assay continued with the Seahorse XF Cell Mito Stress Test Kit in accordance with the manufacturer’s instructions. Measurements were conducted in triplicate following the sequential administration of mitochondrial inhibitors Oligomycin, FCCP, and Rotenone, as specified by established laboratory protocol [15]. Mitochondrial function parameters, such as basal respiration, ATP production rate, proton leak, maximal respiratory capacity, and reserve respiratory capacity, were quantitatively analyzed using Wave Desktop 2.6 software. Data on OCR were systematically captured and documented using the Seahorse Instrument.

### 2.4. Determining Non-Toxic Concentrations of Mitoxantrone on Neural Progenitor Cells

A sub-toxic dosage of the Mitoxantrone drug was determined using the established MTT tetrazolium bromide salt assay. Around 20,000 hNPCs were plated onto a Matrigel-coated 96-well culture plate and maintained in a humidified 5% CO_2_ incubator at 37 °C. The following day, the medium was switched to a basal DMEM/F12 to induce starvation. The cells underwent exposure to escalating concentrations of Mitoxantrone (0.05 µM, 0.10 µM, 0.5 µM, 1.0 µM, 2.0 µM, and 4.0 µM) along with the positive control of DMSO. Viability assessments were conducted at 24-h intervals for up to 96 h. Four hours prior to the completion of each exposure period, 10 µL of the MTT solution (5 mg/mL) was introduced to each well, followed by a further incubation of approximately 4 h at 37 °C in a 5% CO_2_ atmosphere. Post-exposure, the media containing MTT was removed, and the resultant formazan crystals were solubilized using 200 µL of DMSO. The absorbance of each plate was measured at 550 nm using a Synergy HT spectrophotometer (BioTek, Santa Clara, CA, USA). All experimental runs were performed in triplicate with concurrent positive controls. Our study comprised four groups: normal neural progenitor cells (N-hNPCs), mutated neural progenitor cells (M-hNPCs), N-hNPCs + Mitoxantrone drug (1 µM for 24 h) cells, and M-hNPCs + Mitoxantrone drug (1 µM for 24 h) cells.

### 2.5. Transcriptional Studies

(A) Expression profiling of miRNAs

MiRNA expression profiling was conducted using TaqMan human miRNA panel OpenArray plates configured for a 754-target miRNA assay (Thermo Fisher Scientific, Cat# 4470187). Initially, the total RNA was isolated from all four experimental groups using the mirVana miRNA extraction kit (Cat# AM1561; Thermo Fisher Scientific, USA). This was followed by reverse transcription using the TaqMan miRNA reverse transcription kit along with Megaplex Primer Pools (PN 4444750), incorporating stem-loop reverse transcription primers for the 754 miRNAs. Subsequently, the resulting cDNA was subjected to a pre-amplification stage utilizing the TaqMan PreAmp Master Mix and an OpenArray primer pool (Cat # 4444748; Thermo Fisher Scientific, USA). The pre-amplified products were diluted in a four-to-one ratio with a Tris-EDTA buffer and further amplified on Open Array plates targeting the 754 miRNAs using the 12K Flex Real-Time PCR system (Thermo Fisher Scientific). The ΔΔCt method was applied with mammalian U6 snRNA as the internal reference control for relative quantification.

(B) Real-Time PCR

The extracted RNA was subsequently analyzed via real-time PCR for the expression of various genes at the transcriptional level. The RNA’s concentration was determined using a BioSpectrometer (Bio-Spectrometer kinetic Eppendorf, Hemberg, Germany), while its integrity was assessed using agarose gel electrophoresis. Complementary DNA (cDNA) from these RNA samples was synthesized using a high-capacity cDNA reverse transcription kit (Cat # 4368814, Thermo Fisher Scientific, USA), adhering to the provided protocol. The real-time PCR assays utilized PowerUp SYBR Green Master Mix (Thermo Scientific, Waltham, MA, USA). The relative expression levels were calculated using the −ΔΔCt method, employing the housekeeping genes ACTB and GAPDH as internal standards. The specific forward and reverse primers used for the SYBR green-based real-time PCR of various genes are detailed in Supplementary Table S1.

### 2.6. Immunocytochemical (ICC) and Immunoblotting Studies

The hNPCs from all four groups were cultured on 18 mm glass coverslips coated with Matrigel in 12-well plates in preparation for immunocytochemistry analysis. Following the exposure period, cells were fixed using 4% paraformaldehyde (PFA) for twenty minutes at room temperature and then permeabilized with 0.5% Triton X-100 in PBS for 10–15 min using a rocker shaker at room temperature. Cells were rinsed with PBS and then treated with a blocking solution containing 0.2% Triton X-100 and 0.1% bovine serum albumin for one hour at room temperature. Overnight incubation at 4 °C followed, using primary antibodies (Anti-SQSTM1, Anti-LC3-II, Anti-LAMP1, Anti-TDP-43, Anti-HSP70, and Anti-SOD1) relative to the manufacturer-recommended dilutions. Subsequent steps involved washing the cells with PBS and incubating them with secondary antibodies (Alexa Fluor^®^ 488 goat anti-rabbit (1:500) and Alexa Fluor^®^ 564 goat anti-mouse (1:500)) in darkness for 2 h at room temperature. Post-incubation, cells underwent a final PBS wash before being examined under the EVOS™ FL Auto microscope for FITC, Alexa Fluor^®^ secondary antibodies, and DAPI stains. Three random microscopic fields were chosen for each marker to measure the fluorescence intensity using the Image J software version 1.54g (NIH Image), and the results were presented as the absolute fluorescence intensity (AFI) derived from RGB measurements.

The total protein was extracted using a radio-immunoprecipitation Assay (RIPA) cell lysis buffer supplemented with freshly added Protease Inhibitor Cocktail and phosphatase inhibitor cocktail (PhosSTOP) tablets. The extracted protein’s concentration was determined using the Bicinchoninic Acid (BCA) protein assay. Subsequently, 60 μg of protein was loaded and separated on a 15% SDS-PAGE gel. The resolved proteins were then transferred onto a polyvinylidene difluoride (PVDF) membrane (Immobilion-FL PVDF membrane) and were incubated with primary monoclonal rabbit antibody against LC3-B (Cat# ab221794; Abcam, Cambridge, MA, USA) and primary mouse monoclonal β-actin overnight at 4 °C. The membrane was further incubated with a goat anti-rabbit IgG (H + L) Secondary Antibody, Dylight 800, and a goat anti-mouse IgG (H + L) Dylight 680 Secondary antibody. After incubation, the protein bands were developed using the LiCOR Imager system. The density of each protein band was quantified using Alphaease FC version 4.0 software. All experiments were performed in triplicate, and the results are presented as mean ± SD.

### 2.7. Flow Cytometry Analysis of Autophagy

Autophagy was assessed and evaluated using flow cytometry to measure the expression of LC3-II in the different experimental groups, i.e., N-hNPCs, N-hNPCs + Mito, M-hNPCs, and M-hNPCs + Mito, along with a secondary antibody conjugated with Alexa Fluor 488 goat anti-rabbit IgG (H + L). The cells were collected and washed with chilled phosphate-buffered saline (PBS). The cell pellet was fixed and permeabilized using eBiosciences FOXP3/Transcription factor staining buffer set (Cat# 00-5523-00; Thermofisher Scientific, USA), as per the manufacturer’s protocol. After washing the cells with PBS, they were resuspended in 100 μL of diluted primary LC3-II antibody and incubated for 1 h at room temperature. Following another wash with PBS by centrifugation, the cells were suspended in 100 μL of diluted Alexa Fluor 488-conjugated secondary antibodies. The cells were then incubated in the dark at room temperature for 45 min. After adding 500 μL of PBS, the cells were analyzed using a flow cytometer (BD FACS Canto II).

### 2.8. Assessment of ER Stress and Mitochondrial Membrane Potential

Endoplasmic reticulum activity and mitochondrial membrane potential were assessed using ER-Tracker™ Red (BODIPY™ TR Glibenclamide) and JC-1 Dye, respectively. Cells for all four experimental groups were seeded into Matrigel-coated 6-well culture plates at a density of 200,000 cells per well and incubated overnight at 37 °C in a 5% CO_2_ incubator. For the endoplasmic reticulum stress study, after 24 h of exposure, cells were incubated with 1 μM ER-Tracker™ Red in the culture medium for 30 min. Subsequently, cells were washed with PBS and visualized under a fluorescent microscope with an excitation/emission filter of 587/615 nm. To assess changes in mitochondrial membrane potential, after 24 h of exposure, cells were washed with PBS, followed by adding 2 μM of JC-1 dye to each well, and incubated for 15–30 min at 37 °C in a 5% CO_2_ environment. Fluorescence intensity images were captured by the EVOS™ FL Auto imaging system and analyzed for fluorescence intensity using Image J analysis software (NIH).

### 2.9. In Silico Analysis

Identified dysregulated microRNAs (miRNAs) in M-hNPCs were analyzed using the Gene Ontology (GO) annotation feature of the DIANA-miRPath V4.0 tool (http://www.microrna.gr/miRPathv4). This analysis covered various categories, including biological processes (e.g., cellular functions and processes), molecular activities (e.g., enzyme functions and interactions), cellular components (e.g., organelles and cell structures), and KEGG pathways (a collection of pathway maps representing molecular interaction and reaction networks). Additionally, the TargetScan platform was employed to determine the interactions between the identified deregulated miRNAs and the genes found to be deregulated through real-time PCR analysis. This approach allowed for mapping potential regulatory relationships and the functional impacts of the miRNAs on gene expression.

### 2.10. Statistical Analysis

All experiments were conducted in biological replicates, ensuring the data were collected from multiple independent samples to account for biological variability. The results are presented as the mean values accompanied by their respective standard deviations (SD), providing a measure of the data’s dispersion around the mean. Various tests were utilized for statistical comparisons between groups: the two-tailed Student’s *t*-test for comparing two groups, analysis of variance (ANOVA) for comparing multiple groups, and Pearson correlation for assessing the strength and direction of the linear relationship between two variables. Statistical analysis was performed using GraphPad Prism 8.0.2 software (GraphPad Software, La Jolla, CA, USA) (*p* values */# ≤ 0.05; **/## ≤ 0.01; ***/### ≤ 0.001; and ****/#### ≤ 0.0001). The ‘*’ symbol indicates statistical significance with respect to the N-hNPCs group, while the ‘#’ symbol represents a comparison with respect to M-hNPCs.

## 3. Results

### 3.1. Generation of hiPSC Lines Harboring TDP-43 Gene Mutations and Validation of Their Pluripotency

**Figure 1:**
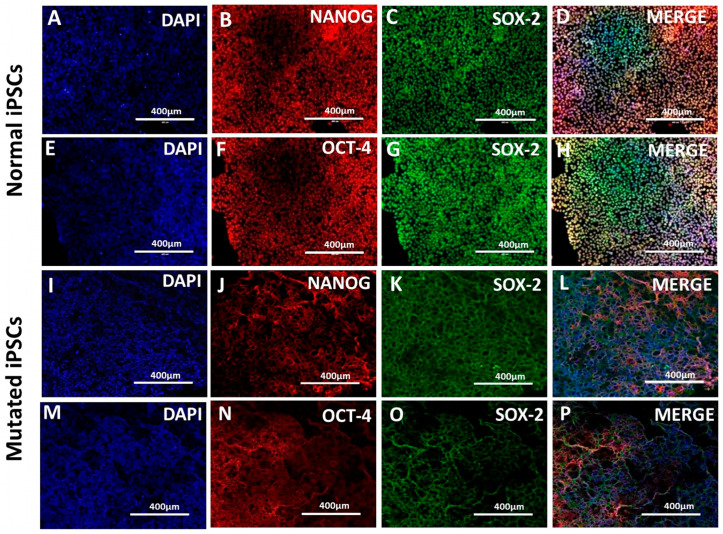
Characterization of the human iPSCs using immunofluorescence. (**A**–**H**) Normal human iPSCs. (**A**,**E**) Nuclear stain–DAPI. (**B**) NANOG (red). (**F**) OCT-4 (red). (**C**,**G**) SOX-2 (green). (**D**,**H**) merge. (**I**–**P**) Mutated human iPSCs. (**I**,**M**) Nuclear stain–DAPI; (**J**) NANOG (red); (**N**) OCT-4 (red); (**K**,**O**) SOX-2 (green); (**L**,**P**) merge. Scale bar 400 µm.

To generate a non-functional variant of the TDP-43 gene, a specific site within this gene was modified to create a mis-functional allele. This modification involved inducing a double-strand break (DSB) at a designated location in the TDP-43 gene, orchestrated by the CRISPR-Cas9 system with a guide RNA (gRNA). The already developed protocol of our laboratory was used to generate the hiPSC lines harboring the TDP-43 gene mutation [10,14]. To check whether mutagenesis influenced hiPSCs pluripotency, N-hiPSCs and M-hiPSCs were investigated for markers such as NANOG, OCT-4, and SOX-2. There was no difference in the expression of pluripotency markers between the N-hiPSCs and M-hiPSCs with TDP43 mutation, showing that the introduced mutations do not affect hiPSC’s pluripotency (Figure 1A–P).

### 3.2. Characterization and Functional Assessment of Mitochondrial Dynamics in Normal and Mutated hiPSC-Derived Neural Progenitor Cells (hNPCs)

**Figure 2:**
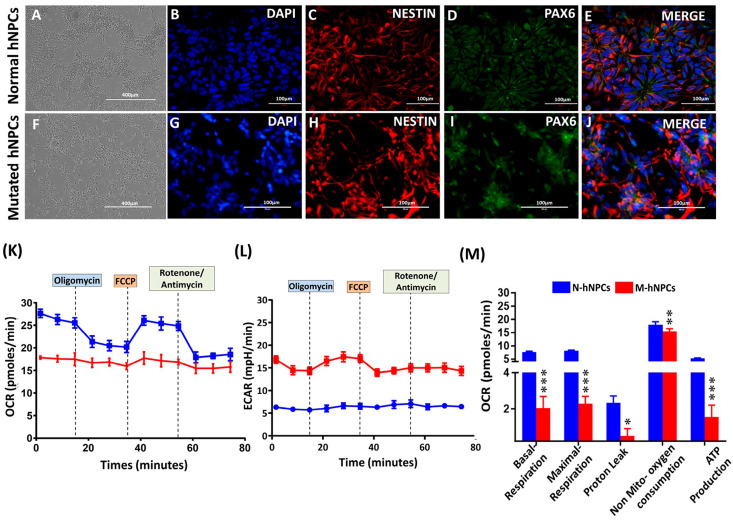
(**A**,**F**) Phase contrast image of normal hNPCs and mutated hNPCs. Characterization of the normal and mutated hNPCs via immunofluorescence. (**B**,**G**) Nuclear stain–DAPI (**C**,**H**) NESTIN (red). (**D**,**I**) PAX6 (green). (**E**,**J**) Merged image of N-hNPCs and M-hNPCs, respectively. (**K**–**M**) Mitochondrial function and cellular bioenergetics parameters were assessed in N-hNPCs and M-hNPCs. The scale bar for (**A**,**F**) is 400 µm, and for (**B**–**E**,**G**–**J**), it is 100 µm. (**K**,**L**) OCR and ECAR profiles of N-hNPCs and M-hNPCs. The ATP synthase inhibitor oligomycin (1 µM), uncoupler FCCP (1 µM), and complex I/complex III inhibitor rotenone/antimycin C (0.5 µM) were injected at the specific time points, as indicated in the figure for analyzing the change in OCR in N-hNPCs and M-hNPCs following the respective drug injection. (**M**) Changes in bioenergetics parameters like basal respiration, maximal respiration, proton leak, non-mitochondrial oxygen consumption, and ATP production in N-hNPCs and M-hNPCs. Data are presented as mean ± SD of the representative readings depicted relative to * *p* < 0.05, ** *p* < 0.01, and *** *p* < 0.001.

Normal and mutant hNPCs were cultured in an hNPC medium (comprising basal DMEM/F12 supplemented with N2 and B27 along with 20 ng/mL human bFGF) and kept at 37 °C in a 5% CO_2_, humidity-controlled incubator. The process of generating hNPCs from hiPSCs involves distinct stages, particularly characterized during the midline stages of hiPSCs transitioning to hNPCs, as detailed in our previous work. This method of hNPC formation follows the protocols established in earlier studies [10,16]. The expression of positive neural progenitor markers PAX-6 and NESTIN indicate that the neural progenitor identity in hNPCs obtained from both N-hiPSC and M-hiPSC sources is almost equal in magnitude (Figure 2A–J).

Flow cytometric analysis revealed that the purity of hiPSC-derived hNPC culture was over @92% (Supplementary Figure S2), where hiPSC-derived hNPCs were tagged with the AF488 (Ex/Em—488/530)-conjugated CD133 and Alexa Fluor 647 (Ex/Em—650/671)-conjugated stage-specific embryonic antigen-4 (SSEA-4) markers.

For the first time, Smith et al. reported that the mutated motor neuron shows a significant drop in the basal [17]. We also assessed the mitochondrial dynamics of N-hNPCs and M-hNPCs using the Seahorse XFp analyzer, and the results are summarized in Figure 2K–M. The sequential addition of Oligomycin A (complex V inhibitor), FCCP (uncoupler), and rotenone (complex I inhibitor) was carried out to determine the effect of mutation on mitochondrial function, as described by the manufacturer. Mutation in the TDP-43 gene significantly decreased the oxygen consumption rate in mutated hNPCs compared to normal hNPCs (27.63 to 17.85 pMol/min) (Figure 2K). Similarly, the extracellular acidification rate (ECAR) significantly changed in mutated hNPCs (Figure 2L). Basal respiration was drastically reduced in the mutated hNPCs compared to normal hNPCs. The other vital parameters, such as proton leak, ATP production reduction, and maximal respiration rate, were also significantly affected in the mutated hNPCs compared to normal hNPCs (Figure 2M).

### 3.3. Analysis of miRNA Expression Profiles and Their Functional Enrichment

**Figure 3:**
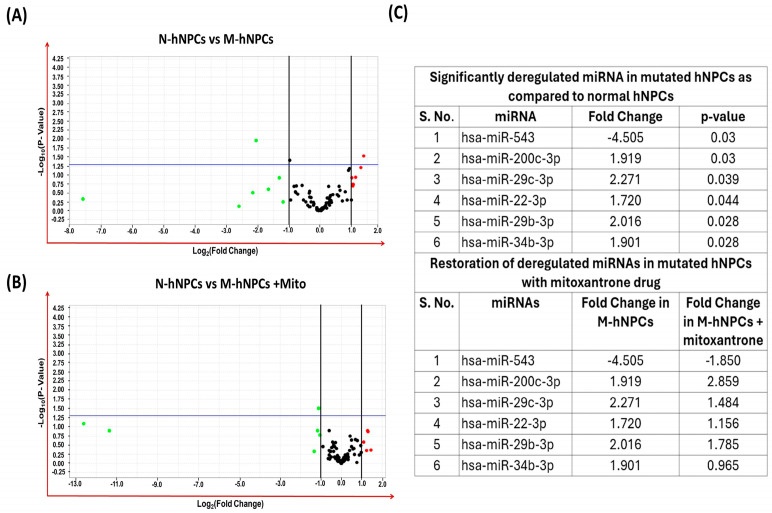
Expression profiling of miRNAs via real-time PCR-based OpenArray. (**A**,**B**) Volcano plot of miRNA expression. The volcano plot is plotted between *p*-value and fold change. The single horizontal line in the volcano plot represents the *p*-value of the *t*-test, a threshold set as 0.05. In contrast, the additional two vertical lines represent the cut-off boundary (±two-fold) for downregulated and upregulated miRNA expression. Above the vertical line, all the green dots and red dots indicate the downregulated and upregulated miRNAs, respectively. (**C**) List of significantly deregulated miRNAs. Expression of miRNAs is represented as the fold change with respect to the normal hNPCs. All the experiments were performed in three biological replicates.

The comprehensive miRNA profiling using the high-throughput OpenArray approach indicated that mutations in the TDP43 gene significantly affect miRNA expression patterns in M-hNPCs relative to N-hNPCs. Analyses involving a volcano plot, constructed from the log2 values of fold change and associated *p*-values, highlighted notable miRNA expression changes in M-hNPCs. Specifically, six miRNAs—miR-543, miR-200c, miR-29c, miR-22c, miR-29b, and miR-34b—were identified as significantly dysregulated in M-hNPCs compared to N-hNPCs. Additionally, the dysregulation of these miRNAs in the M-hNPCs treated with Mitoxantrone was found to be restored, suggesting a compensatory effect of the drug (Figure 3A–C).

To elucidate the functional implications of miRNAs exhibiting significant dysregulation in M-hNPCs, Gene Ontology (GO) enrichment analysis was performed using DIANA-miRPath v4.0. This analysis revealed the involvement of these miRNAs across three key domains: biological processes: cellular protein modification, biosynthesis, Fc-epsilon receptor signaling, neurotrophin–TRK receptor signaling, gene expression, and catabolic processes (Supplementary Figure S5A); molecular functions: ion binding, protein binding, transcription factor activity, nucleic acid binding, enzyme binding, and RNA binding (Supplementary Figure S5B); cellular components: protein complex assemblies, nucleoplasm, cytosol, endoplasmic reticulum lumen, microtubule-organizing centers, and lysosomal lumen (Supplementary Figure S5C).

Additionally, KEGG pathway analysis identified the involvement of these miRNAs in various biological pathways, including Neurotrophin signaling, PI3K-Akt signaling, and axon guidance (Supplementary Figure S5D). The term-centric miRPath module confirmed the correlation between deregulated miRNAs in M-hNPCs and online-identified miRNAs (Supplementary Figure S6A,B).

### 3.4. Gene Expression Analysis

**Figure 4:**
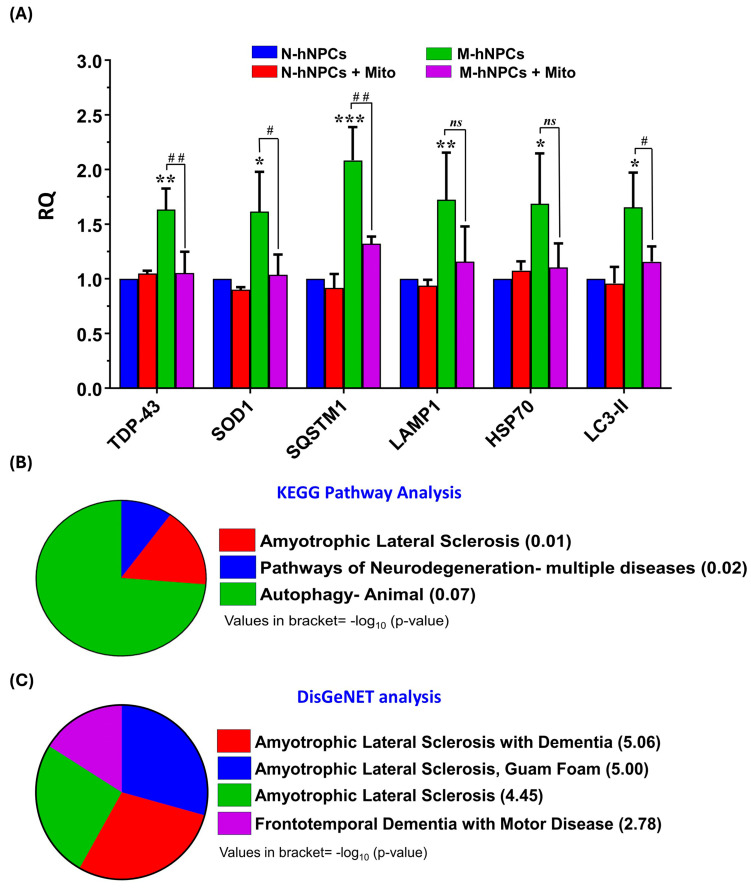
(**A**) Transcriptional changes in the expression level of TDP-43, SOD1, SQSTM1, LAMP1, HSP70, and LC3-II in N-hNPCs and M-hNPCs followed by Mitoxantrone exposure. Bar graphs show the values for different mRNA expressed in relative quantity (RQ). β-Actin was used as an internal control to normalize the data. Data are presented as the ±SD of three separate experiments performed, n = 3 (biological replicates). The changes in expression pattern are statistically significant as indicated by * *p* < 0.05, ** *p* < 0.01, and *** *p* < 0.001 vs. N-hNPCs and # *p* < 0.05 and ## *p* < 0.01; ns = non-significant compared to M-hNPCs exposed to Mitoxantrone. (**B**,**C**) Bioinformatic analysis of the deregulated expression of genes in M-hNPCs. GO enrichment analysis of deregulated genes in the category of the (**B**) KEGG pathway and (**C**) human gene–disease associations category. GO terms with *p*-value < 0.05 are considered significantly enriched and analyzed using the DAVID online tool.

Studies have shown that mutations in the *TDP-43* gene disrupt the regulation of various genes involved in stress granule formation, autogenous processes, and molecular chaperones that clear misfolded proteins [18]. Based on the literature, we conducted the qPCR analysis of genes related to oxidative stress and autogenous processes, such as *TDP-43*, *SOD1*, *SQSTM1*, *LAMP1*, *HSP70*, and *LC3-II*. These genes were found to be upregulated in mutated human neural progenitor cells (hNPCs) compared to normal hNPCs, which is a hallmark of ALS progression. Treating mutated hNPCs with Mito drug downregulated the expression of these genes to basal levels (Figure 4A). Further analysis using the DAVID platform revealed that these genes are associated with various neurodegenerative disorders through KEGG pathway analysis (Figure 4B) and DisGeNET disease association analysis (Figure 4C).

### 3.5. Impact of Mitoxantrone Drug on Autophagy and Stress Granule Markers in M-hNPCs

**Figure 5:**
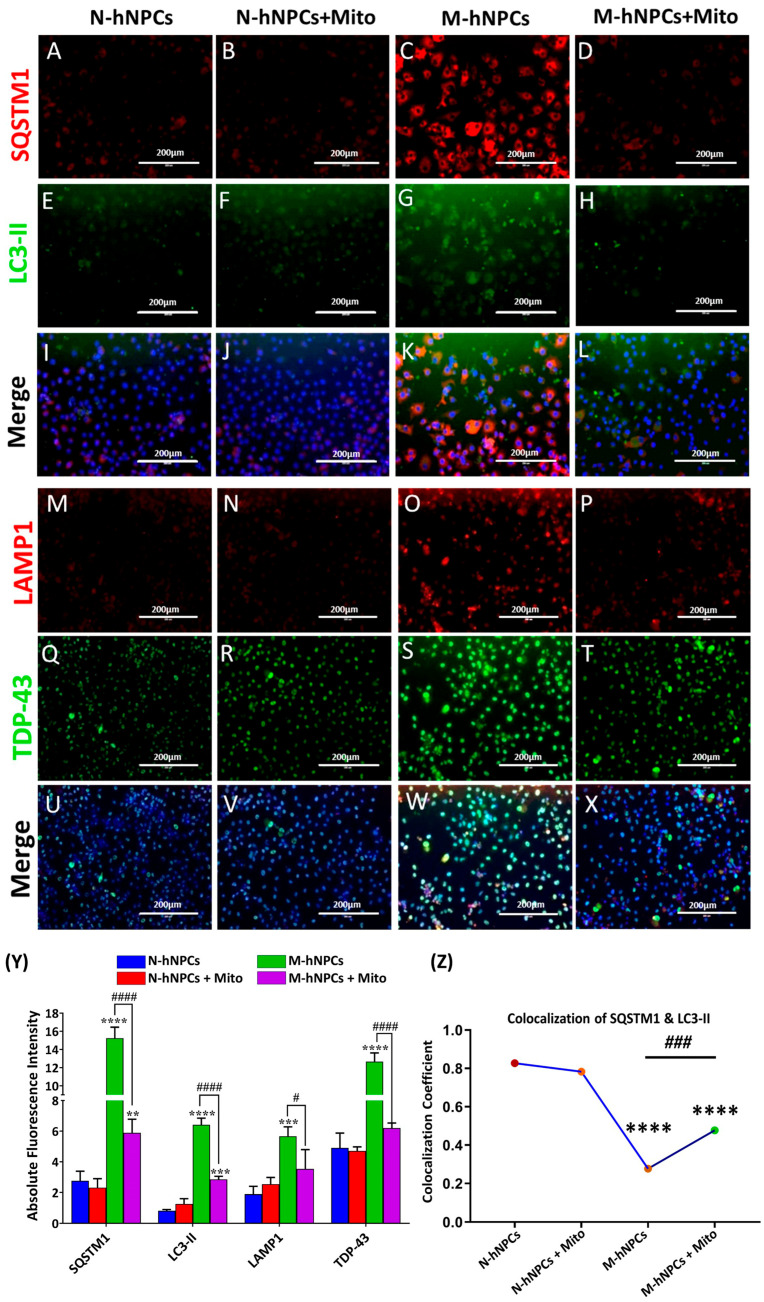
(**A**–**X**) Immunofluorescence images depicting the expression of SQSTM1, LC3-II, LAMP1, and TDP-43 in the four groups (N-hNPCs, N-hNPCs + Mito, M-hNPCs, and M-hNPCs + Mito). (**Y**) Bar graph showing the absolute fluorescence intensity of SQSTM1, LC3-II, TDP-43, and LAMP1 in the experimental groups. (**Z**) Scatter graph showing the colocalization coefficient of SQSTM1 and LC3-II in the four mentioned groups. Data are presented as the ±SD of three separate experiments performed, n = 3 (biological replicates). The changes in expression pattern are statistically significant, as indicated by ** *p* < 0.01, *** *p* < 0.001, and **** *p* < 0.0001 vs. N-hNPCs; # *p* < 0.05, ### *p* < 0.001, and #### *p* < 0.0001 vs. M-hNPCs + Mito. Scale bar 200 µm.

We investigated the changes in autophagy-related markers and markers of defective proteostasis. We examined alterations in autophagy-related markers and indicators of impaired proteostasis. Using immunocytochemistry techniques, we evaluated these markers across all experimental groups, including N-hNPCs and M-hNPCs. Figure 5A–X illustrates each group’s SQSTM1, LC3-II, LAMP1, and TDP43 expression levels. The absolute fluorescence intensity of these markers was quantified and depicted in Figure 5Y. In the M-hNPCs group, the fluorescence intensities for SQSTM1, LC3-II, LAMP1, and TDP43 were 15.3 ± 1.2, 6.4 ± 0.4, 5.7 ± 0.6, and 12.7 ± 1.0, respectively. These values indicate increased stress granule formation and autophagy processes due to the mutation compared to N-hNPCs. Treatment with 1 µg of Mito for 24 h significantly reduced the expression of these markers in the M-hNPCs + Mito group. The mean fluorescence intensities for SQSTM1, LC3-II, LAMP1, and TDP43 in the M-hNPCs + Mito group were 5.9 ± 0.9, 2.9 ± 0.2, 3.5 ± 1.3, and 6.2 ± 0.3, respectively. The fluorescence intensities in the N-hNPCs + Mito group were comparable to those in the N-hNPC group (Figure 5Y). The co-occurrence/co-localization of autophagy gene LC3-II with SQSTM1 was observed to measure the formation of autophagosomes. We performed a colocalization analysis of proteins LC3-II and SQSTM1 to investigate their potential interaction and functional relationship within the cellular environment for the formation of autophagosomes. The co-localization of two proteins such as SQSTM1 and LC3-II was measured in all four groups. The pixel intensity was measured to visualize the co-localization of these two different proteins via Pearson’s coefficient of co-localization (PCC), which presented a significant reduction in the co-localization of SQSTM1 and LC3-II in M-hNPCs (0.276) in comparison to the control hNPCs (0.826). The effect of the exposure of the Mito drug to M-hNPCs (0.476) restores the co-localization of these two proteins with respect to M-hNPCs (Figure 5Z). Overall, our data suggest that even though SQSTM1 and LC3-II expression levels were high in our immunocytochemistry results, neither protein could interact with one another or co-localize in M-hNPCs, which is required to develop a functional autophagosome; thus, the results show failures in autophagy activity in mutated hNPCs.

**Figure 6:**
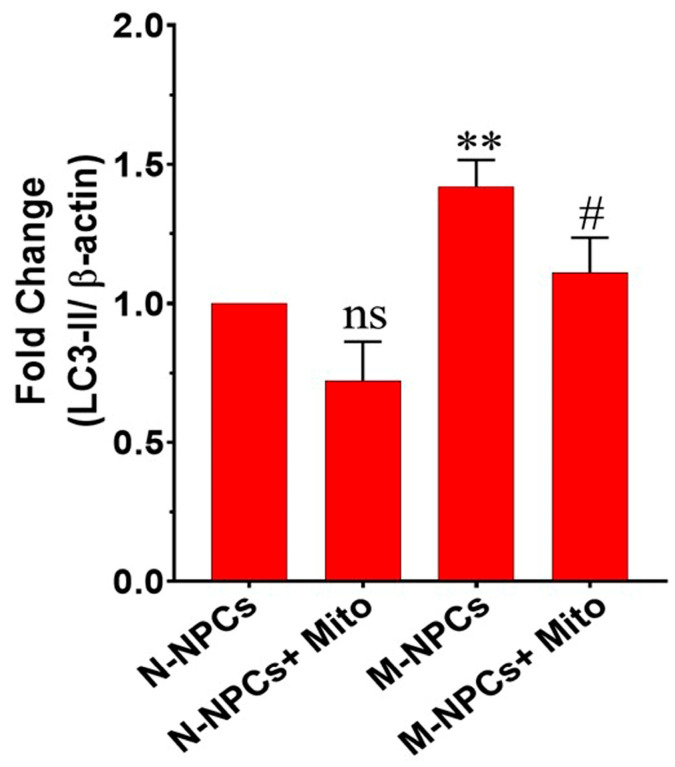
Relative expression of LC3-II protein levels in different groups, i.e., namely, N-hNPCs, N-hNPCs + Mito, M-hNPCs, and M-hNPCs + Mito, were quantified through AlphaeaseFC version 4.0.0 software. The LC3-II levels were normalized to β-actin and plotted as fold change concerning control. The data were represented as the mean ± SD (n = 3 independent experiments), ** *p* < 0.01, with respect to the control, i.e., n-hNPCs; # *p* < 0.05; ns = non-significant with respect to M-hNPCs.

Further, autophagic flux was confirmed with the expression of autophagic marker LC3-II through Western blotting and flow cytometry. The Western blotting data revealed a significant upregulation of LC3-II protein expression in M-hNPCs compared to N-hNPCs (** *p* ≤ 0.01 vs. control). Upon the exposure of Mitoxantrone to M-hNPCs, LC3-II expression was significantly downregulated compared to untreated M-hNPCs (# *p* ≤ 0.05 vs. M-hNPCs). These findings indicate that Mitoxantrone exerts a significant restorative effect on LC3-II expression in M-hNPCs (Figure 6 and Supplementary Figure S3). The flow cytometry results were consistent with the above-mentioned findings, where the LC3-II+ cell population was 0.19% in N-hNPCs and 1.95% in the N-hNPCs + Mito group. In contrast, M-hNPCs demonstrated a significantly higher percentage (71.5%) of the LC3-II+ cell population. In contrast, the number of LC3-II+ cell populations decreased to 39.4% in the M-hNPCs + Mito group, indicating the restorative potential of mitoxantrone. These findings highlight the ability of Mitoxantrone to restore autophagic activity in mutated NPCs partially (Supplementary Figure S4).

**Figure 7:**
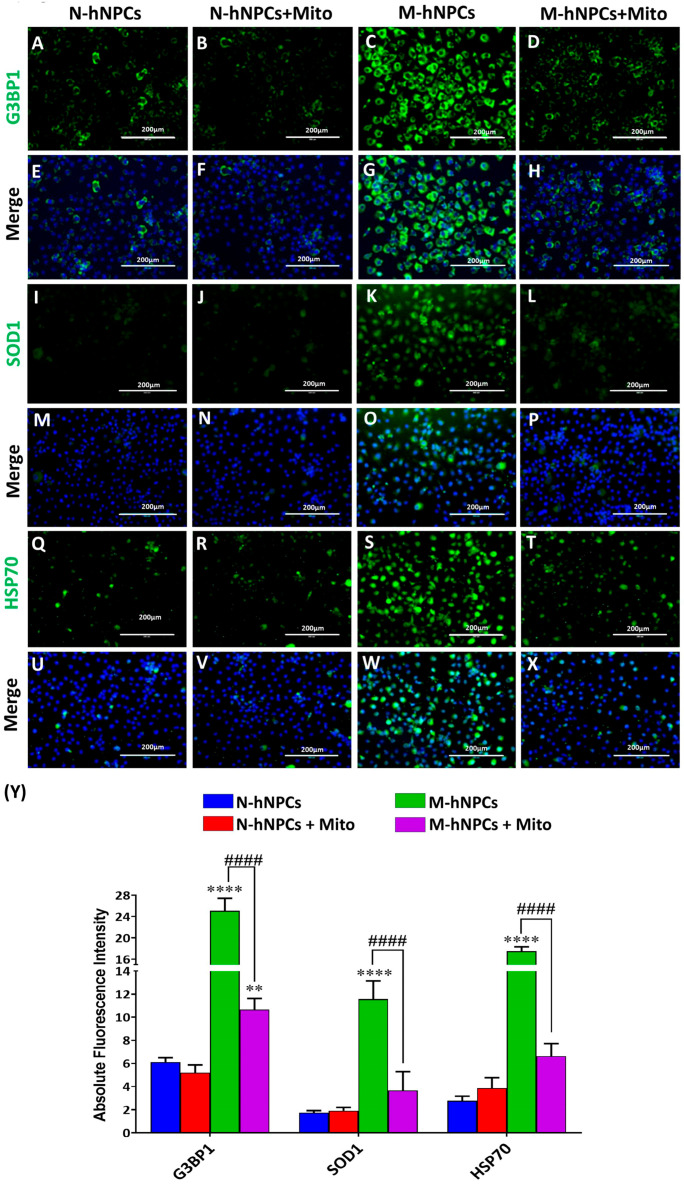
(**A**–**X**) Immunofluorescence images depicting the expression of G3BP1, SOD1, and HSP70 in the four groups (N-hNPCs, N-hNPCs + Mito, M-hNPCs, and M-hNPCs + Mito). (**Y**) Bar graph showing the absolute fluorescence intensity of G3BP1, SOD1, and HSP70 in the four experimental groups. Data are presented as the ±SD of three separate experiments performed, n = 3 (biological replicates). The changes in expression pattern are statistically significant, as indicated by ** *p* < 0.01 and **** *p* < 0.0001 vs. N-hNPCs; #### *p* < 0.0001 vs. M-hNPCs + Mito. Scale bar 200 µm.

We have similarly investigated the expression of stress granules and oxidative stress markers (Figure 7A–X). Our results indicate a significant increase in the expression of G3BP1, SOD1, and HSP70 proteins in M-hNPCs compared to N-hNPCs. Specifically, the absolute fluorescence intensities of G3BP1, SOD1, and HSP70 in M-hNPCs were 25.1 ± 2.4, 11.6 ± 1.6, and 17.5 ± 0.8, respectively. This elevated expression is indicative of ALS progression in neuronal cells. Conversely, N-hNPCs exhibited much lower fluorescence intensities for G3BP1, SOD1, and HSP70, with values of 6.2 ± 0.4, 1.7 ± 0.2, and 2.8 ± 0.4, respectively. Additionally, we evaluated the expression of these markers following exposure to Mito (1 µg for 24 h) in both N-hNPCs and M-hNPCs. Our findings revealed that G3BP1, SOD1, and HSP70 expression was reduced in the M-hNPCs + Mito group (10.7 ± 1.0, 3.7 ± 1.6, and 6.6 ± 1.1) compared to the untreated M-hNPCs group. No significant changes were observed in N-hNPCs exposed to Mito for the same duration (Figure 7Y).

### 3.6. Protective Effects on Mitoxantrone in hNPCs Harboring Mutation-Induced MMP Alterations and ER Stress

**Figure 8:**
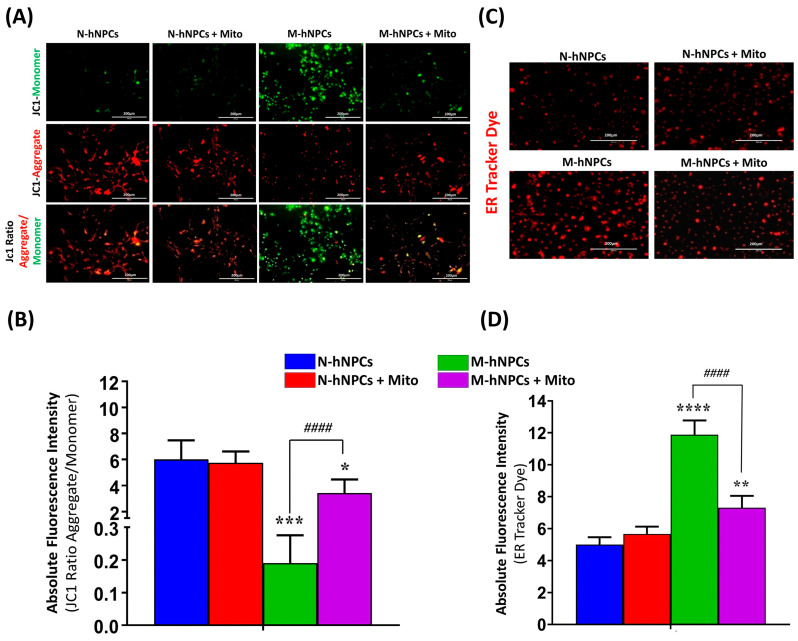
(**A**) Immunofluorescence images of mitochondrial membrane potential (MMP) measurement by expressing JC1 aggregates/monomers. The JC1 aggregates were observed to be red, and monomers were observed to be green. (**B**) Bar graph showing the absolute fluorescence intensity of JC1 aggregates/monomers in all four groups, viz., N-hNPCs, N-hNPCs+ Mito, M-hNPCs, and M-hNPCs+ Mito. (**C**) Expression of ER tracker dye in all four experimental groups. (**D**) Representative bar graphs showing the absolute fluorescence intensity using ER Tracker dye. Data are presented as the ±SD of three separate experiments performed, n = 3 (biological replicates). The changes in expression pattern are statistically significant, as indicated by * *p* < 0.05, ** *p* < 0.01, *** *p* < 0.001, and **** *p* < 0.0001 vs. N-hNPCs; #### *p* < 0.0001 vs. M-hNPCs + Mito. Scale bar 200 µm.

To investigate the protective effect of the Mito drug on mutation-mediated alterations in mitochondrial membrane potential (MMP) and the induction of endoplasmic reticulum (ER) stress in M-hNPCs, JC-1 and ER-Tracker™ Red dye assays were performed as described in the Section 2. JC-1 is excited at approximately 490 nm and emits fluorescence at 530 ± 40 nm for JC-1 monomers (green) and at 580 ± 30 nm for JC-1 aggregates (red). Membrane depolarization is indicated by a higher ratio of monomers to aggregates (530 ± 40: 580 ± 30). We assessed the changes in MMP across all groups after exposure to the Mito drug (1 µg for 24 h), followed by incubation with JC-1 dye. Under normal physiological conditions, mitochondria have a high membrane potential, leading to the formation of JC-1 aggregates (red). In contrast, a lower membrane potential, indicative of mitochondrial dysfunction, forms JC-1 monomers (green). In M-hNPCs, an increase in the JC-1 monomer area and a decrease in the JC-1 aggregate area were observed, signifying MMP loss. This was reflected by the reduced JC-1 aggregate/monomer ratio intensity in M-hNPCs (red: green ratio of 0.2 ± 0.1). Conversely, N-hNPCs exhibited an increased intensity of the JC-1 aggregate/monomer ratio of JC-1 monomers compared to M-hNPCs. The exposure of Mito to N-hNPCs did not significantly alter the MMP (red: green ratio of 5.8 ± 0.9) compared to the untreated N-hNPCs group. However, M-hNPCs treated with Mito showed an increased intensity of the JC-1 aggregate/monomer ratio (red: green ratio of 3.4 ± 1.1) compared to untreated M-hNPCs. These findings suggest that the Mito drug confers protection against mutation-induced MMP disruption in M-hNPCs (Figure 8A,B). We also explored endoplasmic reticulum (ER) stress levels in neural progenitor cells (hNPCs) harboring TDP-43 mutations and assessed the therapeutic efficacy of the Mitoxantrone drug in mitigating this stress. ER stress was evaluated using the ER-Tracker™ Red dye, as depicted in the immunocytochemistry assay (Figure 8C). Live cell imaging revealed that M-hNPCs exhibited a pronounced increase in ER Tracker Red fluorescence and the formation of distinct vesicular structures within the endoplasmic reticulum, indicative of ER stress. Quantitative analyses of the live cell imaging data demonstrated a significant accumulation of ER Tracker Red-positive vesicular structures in M-hNPCs (11.8 ± 0.9) compared to their normal counterparts (N-hNPCs) (5.0 ± 0.5). This suggests an enhancement of ER stress due to the TDP-43 mutation in M-hNPCs. Remarkably, treatment with Mito was found to decrease the expression of the ER Tracker Red dye in M-hNPCs (7.3 ± 0.7) (Figure 8D). These findings suggest that Mitoxantrone may play a role in reducing the aggregation of mutated TDP-43 protein, thereby alleviating ER stress in these cells.

## 4. Discussion

This study explores the potential of Mito in alleviating hyperactive autophagy and lysosomal dysfunction in ALS-linked TDP-43-mutated M-hNPCs using an in vitro model. The model was generated by differentiating normal and mutated hiPSCs into hNPCs, with the mutation introduced using the CRISPR-Cas9 system. Numerous studies have demonstrated the high specificity and efficiency of the CRISPR-Cas9 system in targeting both alleles of a gene [19,20]. Additionally, research has shown that using CRISPR-Cas9 to create missense mutations in TDP-43 does not significantly alter protein expression levels, as measured by various assays such as Western blotting, immunofluorescence, and detergent-soluble assays [21,22]. The Mito drug is a synthetic nucleic acid intercalating molecule that can directly bind to DNA/RNA and inhibit DNA synthesis, DNA repair, and topoisomerase II activity in tumor cells [23,24]. It also serves as an immunomodulatory and immune-suppressive drug to treat multiple sclerosis [25]. Posing the virtue of intercalating with DNA, we hypothesized that it might modulate the production of DNA/RNA binding protein TDP-43 by interfering with the expression of their mutated DNA strand at the transcriptional level. We used TDP-43-mutated hiPSC-derived (M-hNPCs) to investigate the neuroprotective efficacy of Mitoxantrone in mitigating the key pathological hallmark and associated processes against the experimental exposure to a non-cytotoxic dose (1 μM for 24 h) of Mitoxantrone.

Several ALS-linked mutations, including p.M337V in the TDP-43 gene, have been shown to trigger the overexpression of the insoluble form of TDP-43 in neurons, causing cytoplasmic aggregation, dysfunctional mitochondrial and cellular proteostasis systems, axonal neuropathy, abnormal neurites, and decreased cell viability [26,27]. The loss of TDP-43, a DNA/RNA-binding protein, induces extensive cryptic polyadenylation in ALS and frontotemporal dementia (FTD). This phenomenon was observed in a study where researchers found that TDP-43 depletion leads to the inclusion of cryptic exons, resulting in aberrant mRNA transcripts [28].

ALS is a late-onset, progressive-motor-neuron degenerative disease. As time passes, the complete loss of normal TDP-43 expression mimics our developed model system at a very early stage where neural progenitor cells derive the functional motor neuron [29]. Our study utilized TDP-43-mutated hNPCs to study the therapeutic potential of miRNAs and Mitoxantrone drugs. Our real-time PCR and immunocytochemistry-based findings have shown an increased expression/accumulation of mutated TDP-43 protein in M-hNPCs compared to N-hNPCs (Figure 4A). The stress caused by accumulated TDP-43 resulted in an increment of G3BP1-positive stress granules in M-hNPCs compared to normal hNPCs, which may be an early event in gathering insoluble cytoplasmic inclusions [30]. We found a significant decrease in the expression/accumulation of abnormal TDP-43 proteins and G3BP1 in Mito-treated M-hNPCs compared to N-hNPCs. The findings suggest that the DNA intercalating property of Mitoxantrone may be responsible for lowering the mutant TDP-43 expression/aggregates. Studies have also shown that mutated TDP-43 (p.Q331K and p.M337V mutations) might impact mitochondrial dynamics (fusion and fission) and function by colocalizing with mitochondria in axon and skeletal muscle fibers and impairing mitochondrial complex levels, mitochondrial membrane potential, mitochondrial length and density, and their transport in axons in ALS disease conditions [31,32]. Additionally, toxicity due to the gain in the function of the TDP-43 gene significantly impaired the mitochondrial oxygen consumption rate (OCR), ATP production, and the maximal respiratory rate in neurons [33]. To validate the generated CRISPR-Cas9-mediated mutation in the TPD-43 gene in hiPSC-derived hNPCs, we examined these hallmark changes in M-hNPCs. Our results were consistent with previous reports indicating a significant drop in OCR, basal respiration, maximal respiration, and other vital parameters in M-hNPCs (Figure 2K). We also observed a significant drop in mitochondrial membrane potential (MMP) in mutated hNPCs, which signifies the increased depolarization of the mitochondrial membrane, leading to oxidative stress (Figure 2L). A significant increase in achieving the normal levels of MMP in Mito-exposed M-hNPCs indicates the improvement in mitochondrial functions and homeostasis by Mitoxantrone. This indicates Mitoxantrone’s restorative potential in reversing mutation-driven abnormal cellular physiology toward normalcy in M-hNPCs. Cross-seeding behaviors were seen between pathological TDP-43 and SOD1 in cultured neurons. Trist and coworkers (2022) have shown that mutant TDP-43 promotes the disorganization of wild-type SOD1. The structurally disordered (dis)SOD1 becomes mis-localized and deposited with p62/SQSTM1 in motor neurons through an indirectly impaired ER-Golgi trafficking mechanism in the motor neurons of non-SOD1-fALS and sALS patients [34]. In ALS patients, both familial (fALS) and sporadic (sALS), misfolded SOD1 proteins are mislocalized and accumulated with p62/SQSTM1 in motor neurons. This occurs due to impaired ER–Golgi trafficking mechanisms. Additionally, ALS is characterized by the mislocalization of TDP-43 from the nucleus, disrupting RNA processing and impairing critical cellular pathways essential for neuronal health and neuromuscular junction (NMJ) integrity. Mutations in the *TARDBP* gene, which encodes TDP-43, are associated with RNA abnormalities, including altered gene expression, mis-splicing, and decreased transcript stability. TDP-43 regulates alternative exon inclusion as a splicing repressor, and its mutation has been implicated in extensive splicing disruptions. Studies suggest that TDP-43 mutations can dysregulate its expression through feedback mechanisms, leading to upregulation or downregulation [35]. Our study found that the increased expression of TDP-43 in mutated hNPCs is a compensatory mechanism for maintaining normal cellular homeostasis.

It shows that multifaceted interaction and the co-deposition of protein disSOD1, TDP-43, and p62/SQSTM1 in ALS conditions define the severity of ALS-associated pathologies. Our study also explored the accumulation of normal SOD1 in M-hNPCs. We observed significant fold increases in the expression of SOD1, which may be indicative of its mutated TDP-43-driven structural deformation and mislocalization in cells (Figure 7K).

The accumulation of ALS-associated mutant TDP-43 and stress granules has been shown to induce ER stress, which further prompts the cytoplasmic accumulation of mutant TDP-43 and stress granules [36]. The increased expression of ER stress-mediating proteins CHOP, GRP-78, and phospho eIF2α plays a role in neuronal cells overexpressing the mutant TDP-43 [37]. In the present study, ER Tracker Red staining revealed increased ER stress in M-hNPCs compared to N-hNPCs, where the treatment of Mito relative to M-hNPCs significantly reduced ER stress compared to M-hNPCs. Likewise, defects at various stages of autophagy have been associated with the pathological mutations of several ALS-linked genes, including SOD1, p62/SQSTM1, TDP-43, and optineurin, suggesting that such defects may play a causative role in the pathogenesis of this condition [38]. The colocalization of autophagy-related protein LC3-II with SQSTM1 indicates that active autophagy is crucial for the clearance of these aggregates [39]. Our previous study has shown that hyperactivated autophagy as a result of the over-accumulation of LC3 I/II and LAMP1 and the formation of autolysosomes in SOD1-mutated hiPSC-derived motor neurons is a key pathological mechanism in ALS [10]. In the present study, the increased expression of autophagy-associated proteins (LC3-II, SQSTM1, LAMP1, and HSP-70) and the colocalization of LC3-II with mutated SQSTM1 in M-hNPCs clearly showed the occurrence of dysfunctional hyperactivated autophagy driven by TDP-43 accumulation (Figure 5A–Z). The reduction in the expression of these autophagy-related proteins to the level of normal autophagy in Mito-treated mutated hNPCs reveals the therapeutic potency of Mito in mitigating the key pathological mechanisms, ER stress and autophagy, in ALS etiology.

ALS is often associated with mutations in proteins linked to stress granules (SGs). These dynamic condensates form through liquid–liquid phase separation, a process that can become aberrant and contribute to disease phenotypes. Research has identified various small molecules as potential therapeutic agents for ALS [40]. One such molecule is Mitoxantrone, an anthracene-dione antineoplastic agent, which has shown promise as a therapeutic option for ALS. Fang et al. screened approximately 5000 small molecules in HEK293T and neural precursor cells (NPCs) for their effects on stress granule formation, identifying around 100 hits, including Mitoxantrone. This type II topoisomerase inhibitor, approved for leukemia and multiple sclerosis, reduces TDP-43 recruitment to SGs and mitigates persistent cytoplasmic TDP-43 aggregates in induced pluripotent stem-cell-derived motor neurons (hiPSC-MNs) [23]. Additionally, Wheeler et al. found that Mitoxantrone suppresses fused-in-sarcoma (FUS) recruitment to SGs and decreases FUS droplets’ size in vitro. These findings suggest that Mitoxantrone’s therapeutic potential lies in modulating SG composition rather than blocking their formation, addressing key RNA-binding proteins involved in ALS pathology [40]. While Mitoxantrone has shown promise, there is currently no direct approach for treating ALS with this drug. Our study investigated the combined effect of deregulated miRNA with Mitoxantrone treatment in mutated human neural progenitor cells (M-hNPCs).

A promising therapeutic approach for ALS involves using antisense oligonucleotides (ASOs), synthetic nucleotide strands designed to inhibit specific microRNAs (miRNAs). For example, targeting miR-218, which is elevated in ALS and contributes to astrocyte dysfunction, has shown potential in preclinical models in slowing disease progression [41]. Research suggests that ALS-associated proteins, such as FUS and TDP-43, play critical roles in miRNA biogenesis by participating in the Drosha and Dicer/miRNA processing complexes [42]. TDP-43 facilitates the maturation of specific miRNAs by interacting with Drosha and primary miRNA transcripts in the nucleus and with precursor miRNA terminal loops in the cytoplasm, enhancing their processing by the Dicer complex. Additionally, TDP-43 regulates the abundance of the microprocessor complex, influencing the entire miRNA repertoire during in vitro neuronal differentiation [43].

Mitoxantrone, a synthetic anthracenedione, has been found to exhibit antineoplastic and immunosuppressive properties through its ability to intercalate into DNA, inducing crosslinks and strand breaks while also interfering with RNA function and inhibiting topoisomerase II [44]. Notably, its immunosuppressive effects have been leveraged in the treatment of multiple sclerosis (MS), targeting aberrant T- and B-cell responses, myelin damage, and axonal injury within the central nervous system. Recent investigations have explored its potential application in ALS therapy, focusing on its capacity to modulate immune responses and inhibit microglial activation, both critical components of ALS pathology [13].

Furthermore, emerging evidence suggests that Mitoxantrone may interact with microRNA pathways in ALS, although the precise mechanisms underlying this interaction remain to be fully elucidated. Intriguingly, Mitoxantrone has been identified as a stress granule (SG) inhibitor capable of binding directly to RNA and disrupting the accumulation of ALS-associated RNA-binding proteins in SGs [23]. Our studies have corroborated these findings, demonstrating the downregulation of stress granule markers (G3BP1, HSP70, SOD1) following the treatment of mutated human neural progenitor cells (M-hNPCs) with Mitoxantrone (Figure 7). These observations suggest that Mitoxantrone may modulate SG dynamics by interfering with the RNA-dependent assembly of RNA-binding proteins, although further investigation is necessary to elucidate the underlying mechanisms.

Studies have explored the direct and indirect association of mutation in the genes causing ALS with miRNAs [45,46]. Mutant TDP-43 overexpression in motor neuron-like cells promotes stress granule formation and interrupts miRNA biogenesis and miRNA processing [7]. A study based on TaqMan OpenArray miRNA profiling in postmortem human spinal cord tissue from 12 sALS patients revealed that TDP-43 mutation regulates global reduction (98%) in mature miRNA [47]. Studies have shown differentially expressed miRNA (miR-34a, miR-345, miR-200c, and miR-10a) in presymptomatic ALS mutation carriers and symptomatic ALS patients as a diagnostic marker for therapeutic targets in ALS disease [48,49]. A study has shown that the abnormal binding activity of miR-543 leads to the upregulation of autophagy, whereas another study reported the inhibition of autophagy upon the silencing of miR-543 [50,51]. TaqMan OpenArray miRNA profiling revealed the upregulated expression of miR-543 in Parkinson’s disease patients [52]. In the present study, utilizing TaqMan OpenArray miRNA profiling, we also found six miRNAs (miR-543, miR-34a, miR-200c, miR-22, miR-29b, and miR-29c) altered in mutated hNPCs among two (miR-200c and miR-29c) that are already reported in ALS disease. Martinez and Peplow reported that miR-29b, miR-22, miR-200c, and miR-34a were deregulated in the various animal models of ALS [53]. We have also identified these miRNA deregulations in our cellular ALS model (M-hNPCs). The bioinformatic analysis also revealed that these microRNAs are directly involved in ALS progression and autophagy (Supplementary Figure S6A,B). These identified miRNAs may emerge as a mediator for the restorative potential in Mito-treated M-hNPCs. It may be suggested that these miRNAs’ antisense therapeutic molecules could be a potent therapeutic target for ALS management.

## 5. Conclusions

In summary, our research findings indicate that the mutated TDP-43 protein plays a pivotal role in disrupting autophagy, endoplasmic reticulum (ER), and mitochondrial activities in ALS-like pathological M-hNPCs. Notably, the pharmacological drug Mitoxantrone ameliorated ALS-like pathology by inhibiting the accumulation of mutated TDP-43 protein and modulating cellular processes. To the best of our knowledge, this study is the first to elucidate the protective effect of Mitoxantrone, along with the microRNA, in the TDP43-mutated ALS model. Furthermore, our study suggests an interplay between miR-543 and autophagy as an upstream event in the manifestation of ALS pathological features. The identified dysregulated miRNAs like miR-543, miR-29b, miR-22, miR-200c, and miR-34a may serve as potential antisense therapeutic agents, either alone or in combination with mitoxantrone, although further validation is required to substantiate this notion. Our in vitro ALS model provides a valuable platform for investigating the efficacy of various anti-ALS biological therapeutic molecules, warranting further research in this area.

### Future Directions

The findings derived from TDP-43-mutated NPCs, a cellular model of ALS, can be further validated through in vivo preclinical studies. The administration of a calculated equivalent dose of Mitoxantrone in a rodent model of ALS may provide real-time confirmation of its efficacy in mitigating ALS symptoms. Consistent molecular findings and ALS-associated neurobehavioral assessments such as rotarod testing, grip strength analysis, and step length measurements could strengthen the understanding of Mito’s neuroprotective mechanisms. Furthermore, the identified dysregulated miRNAs, which may function as ASOs, could be delivered to targeted brain regions in ALS rodent models, either alone or in combination with Mitoxantrone, in order to evaluate their therapeutic potential and comparative efficacy. The in vivo outcomes may substantiate preliminary findings, facilitating translational refinement and potential progression toward clinical applications in human ALS therapeutics. 

## Data Availability

The raw data are available in the repository of the Institute on its server and with the corresponding author, too.

## Supplementary Materials

The following supporting information can be downloaded at: https://www.mdpi.com/article/10.3390/pharmaceutics17040410/s1, Figure S1. The image illustrates the mechanism of CRISPR-Cas9 gene editing technology. At the top, the CRISPR-Cas9 complex is shown with the Cas9 enzyme guided by a specific guide RNA (gRNA) to a target DNA sequence. The gRNA contains a sequence complementary to the target DNA, enabling precise targeting. The Cas9 enzyme makes a double-strand break (DSB) at the target site adjacent to a protospacer adjacent motif (PAM) sequence, which is recognized as NGG. The bottom part of the image explains the two primary pathways for repairing the DSB: homologous recombination (HR) and non-homologous end joining (NHEJ). In homologous recombination, a donor DNA template is used to repair the break accurately through homology-directed repair, leading to precise gene editing. In non-homologous end joining, the break is repaired without a template, often resulting in insertions or deletions (indels), which can disrupt the target gene (Created through BioRender). Figure S2: Characterization of hiPSC-derived human neural progenitor cells (hNPCs) via flow cytometry. CD133 was used as a positive marker for hNPCs, while SSEA-4 served as a negative marker for NPCs and a positive marker for undifferentiated hiPSCs. Figure S3: Protein expression of LC3-II was assessed in the different experimental groups, namely, N-hNPCs, N-hNPCs + Mito, M-hNPCs, and M-hNPCs + Mito. The LC3-II protein and the β-actin levels were examined through Western blot. The bands of the blots have been cropped with no further manipulation. Figure S4. Analysis of autophagy in different experimental groups using the autophagic marker LC3-II, assessed via flow cytometry. Figure S5: (A–D) Bioinformatic analysis of the deregulated miRNAs in M-hNPCs. GO enrichment analysis of miRNAs is shown in the category of (A) biological process, (B) molecular function, (C) cellular component, and (D) KEGG pathway. The top 15 GO terms with a p-value less than 0.05 are considered significantly enriched and shown in the heat map. Figure S6: (A,B) Heat map of miRNAs involved in various cellular functions and diseased conditions (vice versa) identified via term-centric analysis using the DIANA-miRPath v4.0 online web portal). Table S1: List of primers used in RT-PCR and their sequences. Table S2: Bioinformatic analysis of the deregulated miRNAs in M-hNPCs. GO enrichment analysis of miRNAs is shown in the category of biological processes using the DIANA-miRPath v3.0 online web portal. Table S3: Bioinformatic analysis of the deregulated miRNAs in M-hNPCs. GO enrichment analysis of miRNAs is shown in the category of molecular processes using the DIANA-miRPath v3.0 online web portal. Table S4: Bioinformatic analysis of the deregulated miRNAs in M-hNPCs. GO enrichment analysis of miRNAs is shown in the category of cellular processes using the DIANA-miRPath v3.0 online web portal. Table S5: Bioinformatic analysis of the deregulated miRNAs in M-hNPCs. GO enrichment analysis of miRNAs is shown in the KEGG pathway using the DIANA-miRPath v3.0 online web portal.

## Abbreviations

ALS	Amyotrophic lateral sclerosis
TARDBP	Transactive response DNA-binding protein 43
hNPCs	Human neural progenitor cells
hiPSCs	Human-induced pluripotent stem cells
ER	Endoplasmic reticulum
FDA	Food and Drug Administration
Mito	Mitoxantrone
SOD1	Superoxide dismutase1
MNs	Motor neurons
EB	Embryoid bodies
PFA	Paraformaldehyde
AFI	Absolute fluorescence intensity
RIPA	Radio-immunoprecipitation assay
BCA	Bicinchoninic acid
PVDF	Polyvinylidine difluoride
GO	Gene Ontology
