# Supplementary material for: Targeting TDP-43 Proteinopathy in hiPSC-Derived Mutated hNPCs with Mitoxantrone Drugs and miRNAs
Source: Pharmaceutics. 2025 Mar 25;17(4):410. doi: 10.3390/pharmaceutics17040410 (PMC12030546; doi:10.3390/pharmaceutics17040410)
Supplement: Supplementary file 1 [file pharmaceutics-17-00410-s001.zip › pharmaceutics-3502024-supplementary.pdf]

Targeting TDP-43 proteinopathy in hiPSCs derived mutated hNPCs with Mitoxantrone drug and miRNAs

Uzair A. Ansari 1,†, Ankita Srivastava 1,†, Ankur K. Srivastava 1,†, Abhishek Pandeya 1, Pankhi Vatsa 1,2, Renu Negi 1, Akash Singh 1 and Aditya B. Pant 1,2,*

1 Systems Toxicology Group, FEST Division, CSIR-Indian Institute of Toxicology Research, Vishvigyan Bhawan, 31, Mahatma Gandhi Marg, Lucknow 226001, Uttar Pradesh, India

2 Academy of Scientific and Innovative Research (AcSIR), Ghaziabad 201002, Uttar Pradesh, India

* Correspondence: abpant@iitr.res.in or abpant@rediffmail.com

† These authors contributed equally to this work.

Supplementary Material

1 Supplementary Figures and Tables

1.1 Supplementary Figures

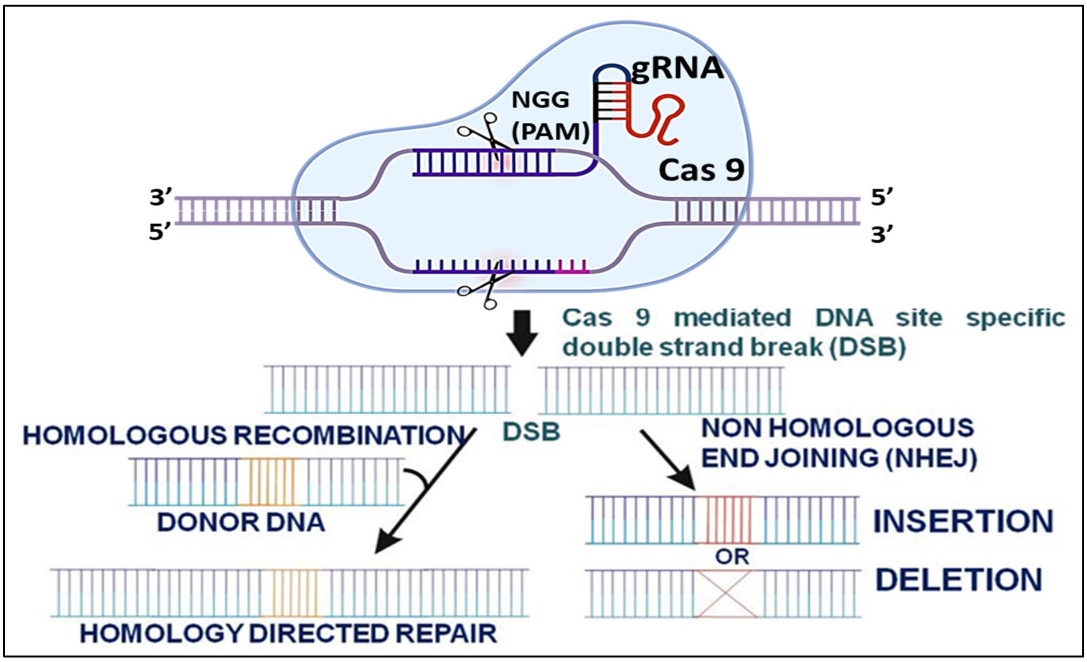

Supplementary Figure S1. The image illustrates the mechanism of CRISPR-Cas9 gene editing technology. At the top, the CRISPR-Cas9 complex is shown with the Cas9 enzyme guided by a specific guide RNA (gRNA) to a target DNA sequence. The gRNA contains a sequence complementary to the target DNA, enabling precise targeting. The Cas9 enzyme makes a double-strand break (DSB) at the target site adjacent to a protospacer adjacent motif (PAM) sequence, which is recognized as NGG. The bottom part of the image explains the two primary pathways for repairing the DSB: homologous recombination (HR) and non-homologous end joining (NHEJ). In homologous recombination, a donor DNA template is used to repair the break accurately through homology-directed repair, leading to precise gene editing. In non-homologous end joining, the break is repaired without a template, often resulting in insertions or deletions (indels), which can disrupt the target gene (Created through BioRender).

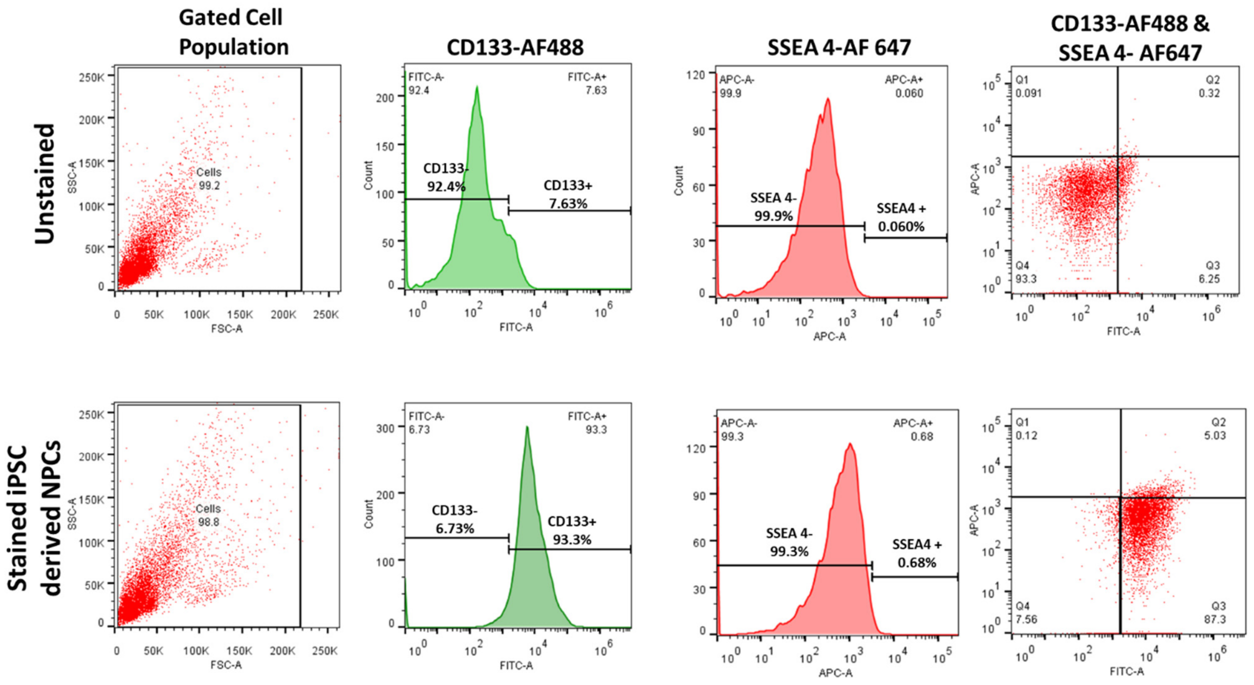

Supplementary Figure S2: Characterization of hiPSC-derived human neural progenitor cells (hNPCs) via Flow Cytometry. CD133 was used as a positive marker for hNPCs, while SSEA-4 served as a negative marker for NPCs and a positive marker for undifferentiated hiPSCs.

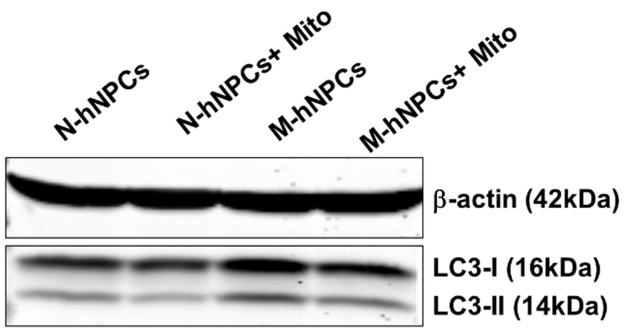

Supplementary Figure S3: Protein expression of LC3-II was assessed in the different experimental groups namely N-hNPCs, N-hNPCs + Mito, M-hNPCs and M-hNPCs + Mito. The LC3-II protein and the β-actin levels were examined through western blot. The bands of the blots have been cropped with no further manipulation.

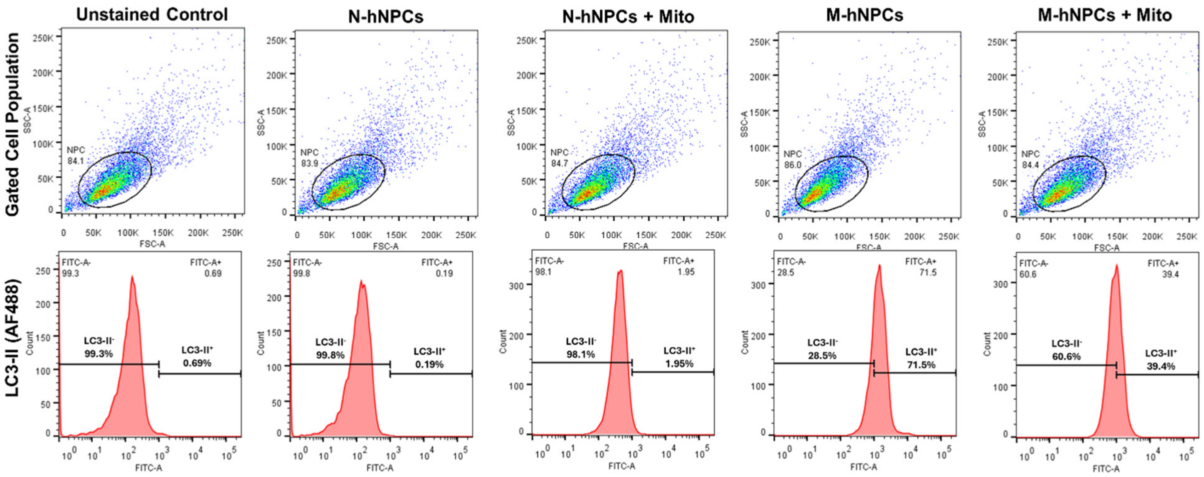

Supplementary Figure S4. Analysis of autophagy in different experimental groups using the autophagic marker LC3-II, assessed *via* flow cytometry.

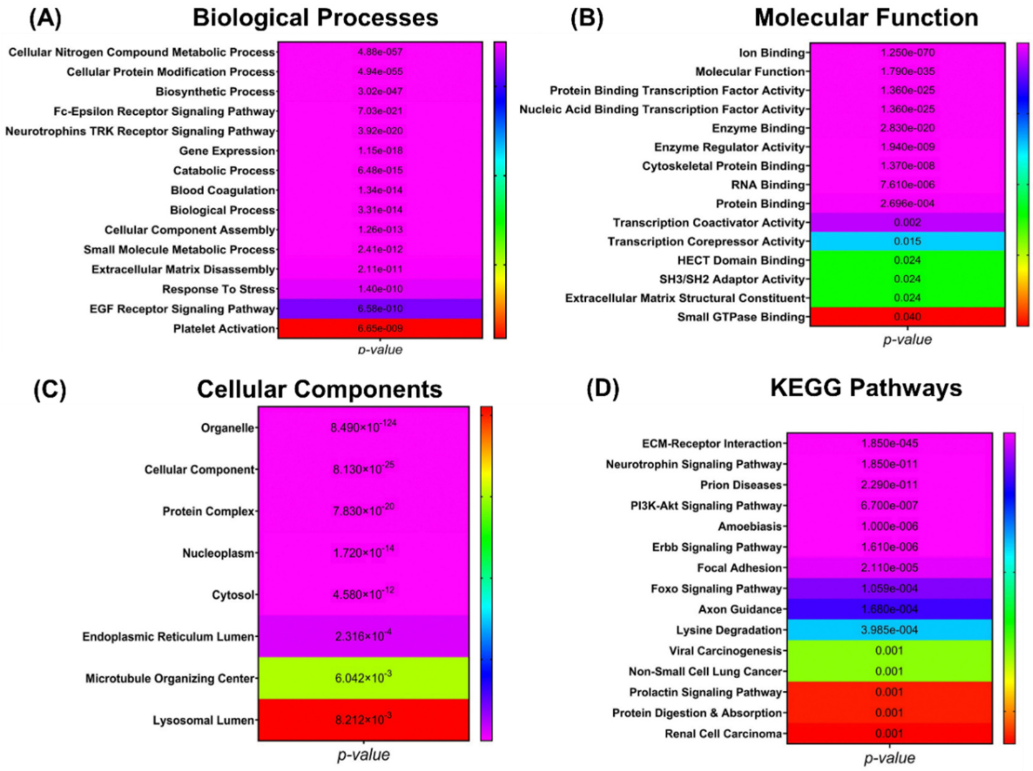

Supplementary Figure S5: (A-D) Bioinformatic analysis of the deregulated miRNAs in M-hNPCs. GO enrichment analysis of miRNAs is shown in the category of **(A)** biological process, **(B)** molecular

Function, **(C)** cellular component **(D)** KEGG Pathway. The top 15 GO terms with a p-value less than 0.05 are considered significantly enriched and shown in the heat map.

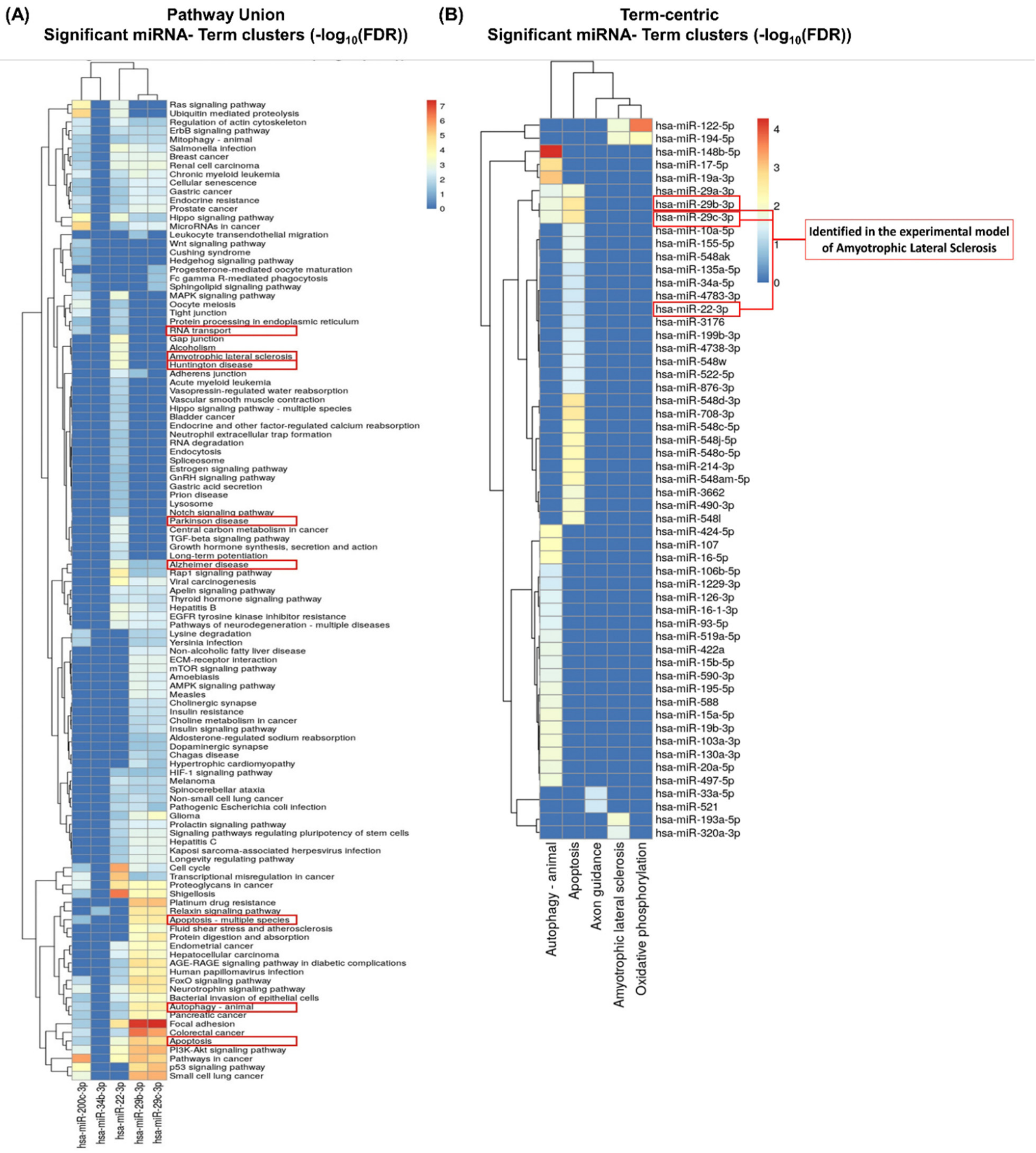

Supplementary Figure S6: (A & B) Heat map of identified miRNAs involved in the various cellular functions and diseased condition and vice-versa identified by term-centric analysis using DIANA-miRPath v4.0 online web portal.

1.2: Supplementary Tables

Supplementary Table S1.

S. No.	Gene Name	Forward Primer 5'→3'	Reverse Primer 5'→3'
1.	TDP-43	AGCAGTCCAGAAAACATCCG	ACCCCTTTGAATGACCAGTC
2.	SQSTM1	AATCAGCTTCTGGTCCATCG	TTCTTTTCCCTCCGTGCTC
3.	LAMP1	GCTCTTCCAGTTCGGGATG	TAGGAATTGCCGACTGTGG
4.	LC3-II	AAGTTCCTTGTACCTGACCATG	CTGAGATTGGTGTGGAGACG
5.	HSP70	AGGACATCAGCCAGAACAAG	CTGGTGATGGACGTGTAGAAG
6.	SOD1	TGTGGCCGATGTGTCTATTG	GCGTTTCCTGTCTTTGTACTTTC

Supplementary Table S1.: List of primers used in RT-PCR and their sequence

Supplementary Table S2.

S. No.	GO Category (Biological)	p-value	Genes	miRNAs
1.	Cellular nitrogen compound metabolic process	4.880E-57	884	6
2.	Cellular protein modification process	4.940E-55	528	6
3.	Biosynthetic process	3.020E-47	768	6
4.	Fc-epsilon receptor signaling pathway	7.030E-21	54	6
5.	Neurotrophin TRK receptor signaling pathway	3.920E-20	71	6
6.	Gene expression	1.150E-18	123	6
7.	Catabolic process	6.480E-15	345	6
8.	Blood coagulation	1.340E-14	102	6
9.	Biological process	3.310E-14	2678	6
10.	Cellular component assembly	1.260E-13	245	6
11.	Small molecule metabolic process	2.410E-12	383	6
12.	Extracellular matrix disassembly	2.110E-11	37	6
13.	Response to stress	1.400E-10	385	6
14.	EGF receptor signaling pathway	6.580E-10	54	6
15.	Platelet activation	6.650E-09	50	6

16.	Cell death	7.260E-09	171	6
17.	Extracellular matrix organization	7.320E-09	87	6
18.	Symbiosis, encompassing mutualism through parasitism	8.030E-09	94	6
19.	Post-translational protein modification	9.010E-09	40	6
20.	Macromolecular complex assembly	9.150E-09	160	6
21.	Viral process	1.160E-08	84	6
22.	Collagen catabolic process	2.500E-08	29	6
23.	Glycosaminoglycan metabolic process	6.390E-08	30	6
24.	Cellular lipid metabolic process	3.600E-07	35	6
25.	Cellular protein metabolic process	4.730E-07	79	6
26.	Phosphatidylinositol-mediated signaling	6.140E-07	37	6
27.	Membrane organization	8.000E-07	105	6
28.	Protein complex assembly	8.520E-07	138	6
29.	Fibroblast growth factor receptor signaling pathway	1.130E-06	46	6
30.	Fc-gamma receptor signaling pathway involved in phagocytosis	2.300E-06	20	6
31.	Mitotic cell cycle	3.130E-06	68	6
32.	Intrinsic apoptotic signaling pathway	3.840E-06	23	5
33.	Axon guidance	4.150E-06	96	6
34.	Transcription initiation from RNA polymerase II promoter	4.310E-06	51	6
35.	Toll-like receptor TLR1:TLR2 signaling pathway	9.140E-06	18	6
36.	Toll-like receptor TLR6:TLR2 signaling pathway	9.140E-06	18	6
37.	Toll-like receptor 10 signaling pathway	1.540E-05	17	6
38.	Transcription, DNA-template	2.320E-05	421	6
39.	Positive regulation of protein insertion into mitochondrial membrane involved in apoptotic signaling pathway	2.980E-05	12	5
40.	Nervous system development	3.870E-05	93	6
41.	Synaptic transmission	4.560E-05	77	6
42.	Cell junction organization	8.140E-05	35	6
43.	Cell motility	8.810E-05	104	6

44.	Toll-like receptor 5 signaling pathway	1.085E-04	17	6
45.	Toll-like receptor 9 signaling pathway	1.085E-04	18	6
46.	Cell junction assembly	1.521E-04	17	6
47.	TRIF-dependent toll-like receptor signaling pathway	1.521E-04	17	6
48.	Myd88-independent toll-like receptor signaling pathway	8.194E-04	17	6
49.	Toll-like receptor 2 signaling pathway	8.194E-04	18	6
50.	Insulin receptor signaling pathway	8.335E-04	37	6
51.	Protein N-linked glycosylation via asparagine	1.334E-03	22	6
52.	Toll-like receptor 3 signaling pathway	1.366E-03	18	6
53.	Nucleobase-containing compound catabolic process	1.367E-03	136	6
54.	Chondroitin sulfate metabolic process	1.394E-03	13	5
55.	Phosphatidylinositol biosynthetic process	1.477E-03	17	6
56.	Chromatin organization	1.477E-03	28	6
57.	Sulfur compound metabolic process	1.477E-03	52	6
58.	Inositol phosphate metabolic process	1.558E-03	12	5
59.	Positive regulation of transcription	1.986E-03	169	6
60.	DNA metabolic process	3.233E-03	123	6
61.	Platelet degranulation	3.755E-03	16	6
62.	Cellular component movement	5.619E-03	24	6
63.	Cell-cell signaling	6.374E-03	104	6
64.	Stress-activated MAPK cascade	7.791E-03	13	6
65.	Regulation of transcription from RNA polymerase II promoter in response to hypoxia	8.555E-03	8	5
66.	Regulation of RNA biosynthetic process	9.570E-03	18	6
67.	Phospholipid metabolic process	9.632E-03	32	6
68.	Toll-like receptor 4 signaling pathway	9.986E-03	19	6
69.	Chondroitin sulfate catabolic process	1.145E-02	6	3
70.	Transcription from RNA polymerase II promoter	1.185E-02	111	6
71.	Leukocyte migration	1.251E-02	23	6

72.	Regulation of small GTPase-mediated signal transduction	1.287E-02	41	6
73.	Myd88-dependent toll-like receptor signaling pathway	1.514E-02	19	6
74.	Cellular component disassembly involved in the execution phase of apoptosis	1.852E-02	10	5
75.	Keratan sulfate catabolic process	2.143E-02	5	4
76.	Sphingolipid biosynthetic process	2.143E-02	12	5
77.	Activation of signaling protein activity involved in unfolded protein response	2.211E-02	13	6
78.	Cell-cell junction organization	2.211E-02	18	6
79.	Cytoskeleton organization	2.540E-02	109	6
80.	Hexose transport	2.650E-02	9	6
81.	Keratan sulfate metabolic process	2.732E-02	8	5
82.	Histone acetylation	2.944E-02	17	5
83.	Androgen receptor signaling pathway	3.239E-02	15	6
84.	Toll-like receptor signaling pathway	3.373E-02	20	6
85.	Xenobiotic metabolic process	3.373E-02	24	6
86.	Immune system process	3.668E-02	232	6
87.	NLS-bearing protein import into the nucleus	3.773E-02	7	5
88.	Positive regulation of transcription from RNA polymerase II promoter	3.893E-02	244	6
89.	Positive regulation of signal transduction	4.237E-02	18	5
90.	Mitotic nuclear envelope disassembly	4.355E-02	8	6
91.	Activation of MAPK activity	4.627E-02	20	6
92.	Response to interleukin-1	4.779E-02	15	6

Supplementary Table S2: Bioinformatic analysis of the deregulated miRNAs in M-hNPCs. GO enrichment analysis of miRNAs are shown in the category of biological process using a DIANA-miRPath v3.0 online web portal.

Supplementary Table S3.

S. No.	GO Category (Molecular)	p-value	Genes	miRNAs
1.	Ion Binding	1.25E-70	1142	6
2.	Molecular Function	1.79E-35	2810	6
3.	Protein Binding Transcription Factor Activity	1.36E-25	136	6
4.	Nucleic Acid Binding Transcription Factor Activity	1.36E-25	231	6
5.	Enzyme Binding	2.83E-20	268	6
6.	Enzyme Regulator Activity	1.94E-09	162	6
7.	Cytoskeletal Protein Binding	1.37E-08	148	6
8.	RNA Binding	7.61E-06	311	6
9.	Protein Binding, Bridging	2.70E-04	38	6
10.	Transcription Coactivator Activity	2.05E-03	72	6
11.	Transcription Corepressor Activity	1.45E-02	55	6
12.	HECT Domain Binding	2.42E-02	3	1
13.	SH3/SH2 Adaptor Activity	2.42E-02	17	5
14.	Extracellular Matrix Structural Constituent	2.42E-02	25	6
15.	Small GTPase Binding	4.00E-02	7	5

Supplementary Table S3: Bioinformatic analysis of the deregulated miRNAs in M-hNPCs. GO enrichment analysis of miRNAs is shown in the category of molecular process using a DIANA-miRPath v3.0 online web portal.

Supplementary Table S4.

S. No.	GO Category (Cellular)	p-value	Genes	miRNAs
1.	Organelle	8.49E-124	1819	6
2.	Cellular component	8.13E-25	2801	6
3.	Protein complex	7.83E-20	676	6
4.	Nucleoplasm	1.72E-14	234	6
5.	Cytosol	4.58E-12	475	6
6.	Endoplasmic reticulum lumen	2.32E-04	42	5
7.	Microtubule organizing centre	6.04E-03	82	6
8.	Lysosomal lumen	8.21E-03	15	6

Supplementary Table S4: Bioinformatic analysis of the deregulated miRNAs in M-hNPCs. GO enrichment analysis of miRNAs is shown in the category of cellular process using a DIANA-miRPath v3.0 online web portal.

Supplementary Table S5.

S. No.	GO Category (KEGG Pathway)	p-value	Gene	miRNAs
1.	ECM-receptor interaction	1.85E-45	29	6
2.	Neurotrophin signaling pathway	1.85E-11	64	6
3.	Prion diseases	2.29E-11	5	5
4.	PI3K-Akt signaling pathway	6.70E-07	26	6
5.	Amoebiasis	1.00E-06	34	6
6.	ErbB signaling pathway	1.61E-06	36	6
7.	Focal adhesion	2.11E-05	69	6
8.	FoxO signaling pathway	1.06E-04	28	6
9.	Axon guidance	1.68E-04	39	5
10.	Lysine degradation	3.98E-04	13	6
11.	Viral carcinogenesis	7.92E-04	47	6
12.	Non-small cell lung cancer	7.92E-04	22	6
13.	Prolactin signaling pathway	1.09E-03	25	6
14.	Protein digestion and absorption	1.09E-03	33	6
15.	Renal cell carcinoma	1.12E-03	44	6
16.	Transcriptional mis-regulation in cancer	1.12E-03	51	6
17.	Small cell lung cancer	1.12E-03	32	6
18.	Glioma	1.22E-03	93	6
19.	Proteoglycans in cancer	1.73E-03	40	6
20.	Choline metabolism in cancer	1.86E-03	36	6
21.	Biotin metabolism	2.01E-03	1	1
22.	Ras signaling pathway	2.54E-03	60	6
23.	mTOR signaling pathway	2.54E-03	24	6
24.	Prostate cancer	2.56E-03	32	6
25.	Long-term depression	3.24E-03	21	6
26.	Platelet activation	5.25E-03	39	6
27.	MAPK signaling pathway	6.38E-03	70	6

28.	Endometrial cancer	8.31E-03	18	6
29.	TGF-beta signaling pathway	8.83E-03	24	5
30.	Bacterial invasion of epithelial cells	8.83E-03	24	6
31.	Estrogen signaling pathway	9.15E-03	27	6
32.	p53 signaling pathway	1.43E-02	24	6
33.	Hippo signaling pathway	1.65E-02	35	5
34.	Phosphatidylinositol signaling system	1.66E-02	25	6
35.	Rap1 signaling pathway	2.14E-02	56	6
36.	Melanoma	2.29E-02	23	5
37.	Chronic myeloid leukemia	2.29E-02	24	6
38.	Signaling pathways regulating pluripotency of stem cells	2.47E-02	40	6
39.	T cell receptor signaling pathway	2.74E-02	31	6
40.	Pancreatic cancer	3.04E-02	19	5
41.	B cell receptor signaling pathway	3.04E-02	23	6
42.	Regulation of actin cytoskeleton	3.13E-02	58	6
43.	Hepatitis B	3.13E-02	39	6
44.	VEGF signaling pathway	4.23E-02	20	5
45.	Dorso-ventral axis formation	4.30E-02	11	6
46.	Glycosaminoglycan biosynthesis - chondroitin sulfate / dermatan sulfate	4.84E-02	4	3
47.	Glycosaminoglycan biosynthesis - heparan sulfate/heparin	4.84E-02	6	4
48.	Sphingolipid signaling pathway	4.84E-02	30	5
49.	Thyroid hormone signaling pathway	4.95E-02	25	6

Supplementary Table S5: Bioinformatic analysis of the deregulated miRNAs in M-hNPCs. GO enrichment analysis of miRNAs is shown in the KEGG Pathway using a DIANA-miRPath v3.0 online web portal.
